# Electrochemical intercalation of anions into graphite: Fundamental aspects, material synthesis, and application to the cathode of dual‐ion batteries

**DOI:** 10.1002/open.202300244

**Published:** 2024-03-01

**Authors:** Yoshiaki Matsuo, Akane Inoo, Junichi Inamoto

**Affiliations:** ^1^ University of Hyogo 13-71 Kitaojicho Akashi Japan

**Keywords:** Electrochemical intercalation, graphite, dual-ion battery, graphite oxide

## Abstract

In this review, fundamental aspects of the electrochemical intercalation of anions into graphite have been first summarized, and then described the electrochemical preparation of covalent‐type GICs and application of graphite as the cathode of dual‐ion battery. Electrochemical overoxidation of anion GICs provides graphite oxide and covalent‐fluorine GICs, which are key functional materials for various applications including energy storage devices. The reaction conditions to obtain fully oxidized graphite has been mentioned. Concerning the application of graphite for the cathode of dual‐ion battery, it stably delivers about 110 mA h g^−1^ of reversible capacity in usual organic electrolyte solutions. The combination of anion and solvent as well as the concentration of the anions in the electrolyte solutions greatly affect the performance of graphite cathode such as oxidation potential, rate capability, cycling properties, etc. The interfacial phenomenon is also important, and fundamental studies of charge transfer resistance, anion diffusion coefficient, and surface film formation behavior have also been summarized. The use of smaller anions, such as AlCl_4_^−^, Br^−^ can increase the capacity of graphite cathode. Several efforts on the structural modification of graphite and development of electrolyte solutions in which graphite cathode delivers higher capacity were also described.

## Graphical abstract

This article first describes fundamental aspects of the electrochemical intercalation of anions into graphite. Then, the electrochemical preparation of covalent‐type graphite intercalation comopouds, especially, graphite oxide and application of graphite as the cathode of dual‐ion battery are discussed.

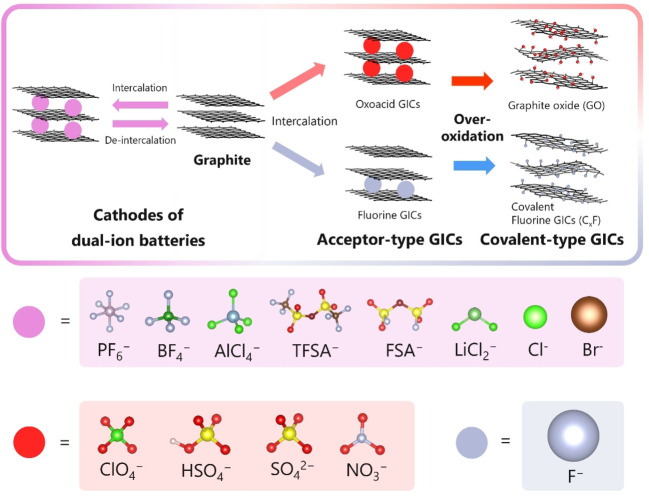

## 1 Introduction

Highly conductive and light weight graphite is one of the most favorable layered host materials for the storage of ions. Large amounts of both anions and cations can enter the interlayer space of it upon electrochemical oxidation and reduction, respectively. Therefore, it is used as the active materials of various types of batteries. Most successful example is the use of it as an anode of lithium‐ion battery. It can deliver a high capacity of 372 mAh g^−1^ with a stoichiometry of LiC_6_ and shows superior cycle life. Recently, graphite has been considered as the alternative of the cathode materials of lithium‐ion batteries in which expensive transition metals such as cobalt and nickel are included. The resulting battery is called as “dual‐ion” or “dual‐carbon” battery, in which anions and cations intercalate/de‐intercalate into/from cathode and anode, respectively. After the pioneering studies by McCullough et al.,[^1^, ^2^] Carlin et al.,[^3^, ^4^] and Santhanam et al.,[^5^, ^6^] a number of papers on dual‐ion batteries (DIBs) using graphite as a cathode material has been recently published. During charging, graphite is electrochemically oxidized, and intercalation of anions occurs, while during discharging, they are de‐intercalated. The used anions are PF_6_^−^, BF_4_^−^, ClO_4_^−^, bis(trifluoromethanesulfonyl)amide (hereafter TFSA, or also abbreviated as TFSI), etc. and they are dissolved in organic solvents such as ethyl methyl carbonate, etc. More recently, ionic liquids or “water‐in‐salt” electrolytes are also used because of their wide electrochemical windows and large concentration of both cations and anions. The advantages of graphite cathode are environmental friendliness, high operating voltage, etc. The other anode materials such as Al, Ca, Mg, Zn, etc. with larger theoretical capacities have been tested instead of graphite anode and charge carriers such as abundantly produced Na^+^ and K^+^ ions can be also used instead of Li^+^.

**Figure 1:**
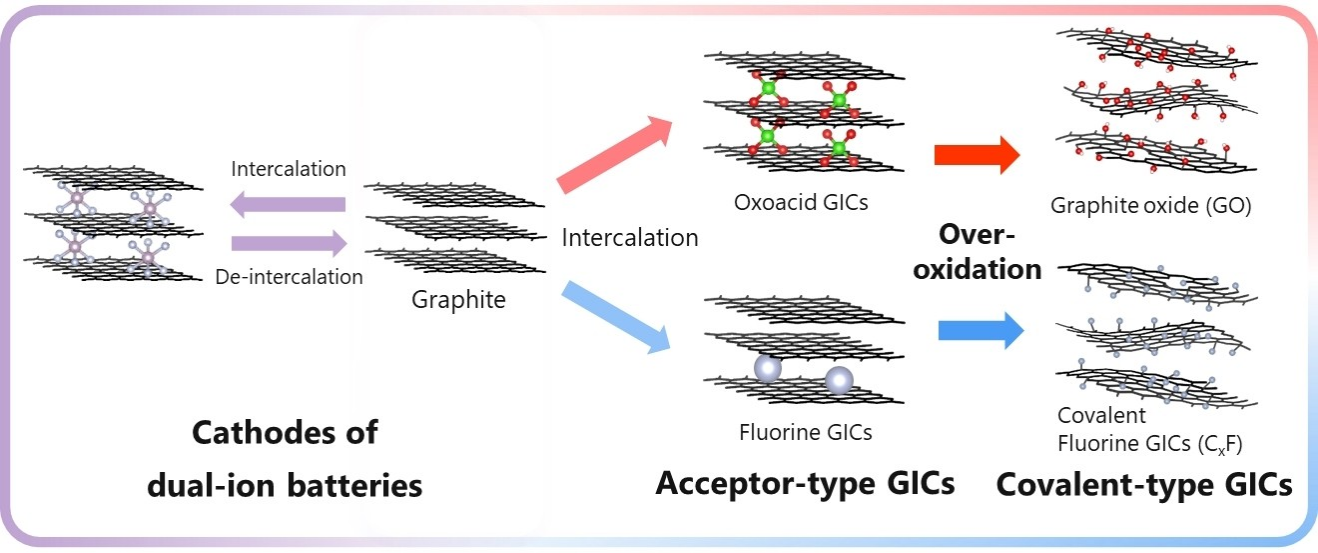
Application of graphite as a cathode of dual‐ion batteries and preparation of covalent‐type graphite intercalation compounds.

Therefore, various types of batteries have been proposed depending on the combination of anodes, electrolytes, and cathodes. Apart from the application to the cathode of DIBs, anion‐GICs are utilized in different manners. Among them, electrochemical production of graphene oxide/graphite oxide (abbreviated as GO) has recently attracted much attention. As described later, GO is classified as covalent type graphite intercalation compound and is a key material as a precursor of graphene. On the other hand, it has recently been reported that the intercalation of anions at high potentials into carbon materials used as a conductive additive in the cathode of LIBs causes degradation.[^7^, ^8^] Therefore, fundamental understanding of anion intercalation reactions into graphite and other carbon materials is critically important not only for DIBs but also for various applications. Several well‐summarized review papers on DIBs have been recently published.[^9^, ^10^, ^11^, ^12^, ^13^, ^14^, ^15^, ^16^, ^17^, ^18^, ^19^, ^20^, ^21^] However, it seems still necessary to overview the related research based on the fundamental properties of graphite intercalation compounds including those studied previously. In this review, therefore, fundamental aspects of the electrochemical intercalation of anions into graphite are first summarized, and then the preparation of covalent‐type intercalation compounds including GO and the electrochemical performance of graphite cathode are described as summarized in Figure 1.

## 2 Graphite intercalation compounds

**Figure 2:**
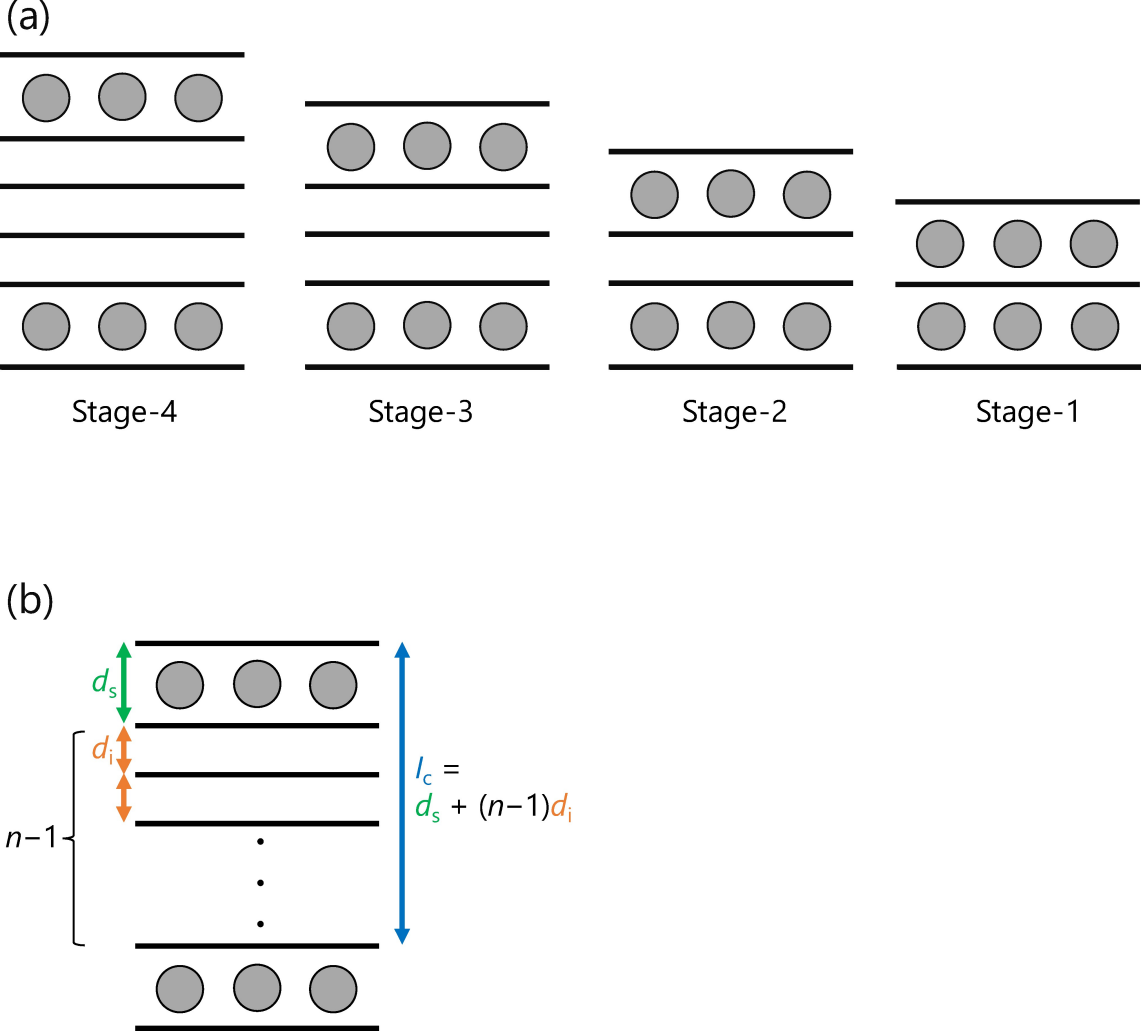
(a) Stage structure of graphite intercalation compounds and (b) their structural parameters.

As already well known, graphite intercalation compounds (GIC) are formed by the insertion of chemical species “intercalate” (formerly called “intercalant”) between the graphene layers of graphite as shown in Figure 2(a). The most important structural feature of GIC is the staging phenomenon. The intercalate layers are periodically arranged in a matrix of graphene layers. This structural regularity is represented by the stage number, i. e., the number of graphene layers between two intercalate layers. The most effective method for determining the stage number is X‐ray diffraction (XRD) measurement. There are several valuable literatures on the determination procedure of the stage number of typical GICs and their structure.[^21^, ^22^, ^23^, ^24^, ^25^] Therefore, in this chapter, the general method of determining the stage number of GICs is first briefly described, followed by the essence of the case of anion intercalation. Thereafter, synthesis methods, structural characteristics, and chemical composition of them are outlined.

### 2.1 Structure analysis of graphite intercalation compounds

The structure of the GIC as represented in Figure 2(b) is considered. Let *n* be the stage number, *d*_init_ the interlayer distance of graphite (0.3354 nm for graphite with high crystallinity) and *d*_sandwich_ the interlayer distance with intercalate, which is called sandwich thickness or gallery height. The repetition distance along the *c*‐axis *I*_c_, which is corresponding to the spacing between adjacent (001) planes if difference of the stacking is neglectable, is expressed as:
(1)

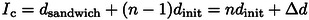




Where Δ*d* represents the increase of interlayer distance by intercalation,
(2)

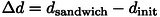




When XRD measurement is performed on the GICs, several 00*ℓ* diffraction peaks are observed depending on the periodicity in the *c*‐axis direction. In particular, 002 peak of pristine graphite gradually shifts towards lower angles with intercalation. To distinguish this peak from other peaks, it is here referred to as “main peak”. Although there are some exceptions, such as tetrachloroaluminate, where the intensity of the main peak decreases largely with anion intercalation, in most cases the main peak remains relatively strong after the shift. Therefore, it is appropriate to focus on this peak to begin with. In addition, most of the anions commonly used for DIB have Δ*d* in the range *d*_init_<Δ*d*<2*d*_init_. For example, PF_6_^−^ (Δ*d*=0.77~0.80 nm),[^26^, ^27^] TFSA^−^ (Δ*d*=0.79~0.81 nm),^[28]^ bis(fluorosulfonyl)amide (FSA^−^) (Δ*d*=ca. 0.78 nm),[^29^, ^30^] ClO_4_^−^ (Δ*d*=0.79~0.80 nm),^[26]^ etc. In this case, the relation *ℓ*=*n*+1 holds between the Miller index *ℓ* of the main peak and the stage number *n*. Using this relationship, the main peak is found to be 00*n*+1 diffraction peak. However, it is difficult to determine only one *n* from the main peak alone, therefore it is necessary to focus on the positions of the other diffraction peaks. To determine *n*, it is necessary to focus on the positions of other diffraction peaks. From Bragg's equation, the relationship between *I*_c_ and *ℓ* and *θ*_00*ℓ*_ is:
(3)

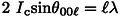




Where *λ* is wavelength of X‐ray. By applying equation (3) to the main peak to obtain *I*_c_, the *θ*_00*ℓ*_ of the other 00*ℓ* diffraction peaks can be calculated from equation (3). By checking whether the calculated peak position of other 00*ℓ* peaks matches the peak positions in experimental XRD pattern, it is possible to determine *n*.

More recently, a simpler method using the following equation which is variant of equation (3) is also widely used:[^31^, ^32^]
(4)

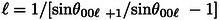




From this equation, the value of *ℓ* of the main peak can be calculated from the peak position of the main and its neighboring peak positions, and then *n* is obtained from *ℓ*=*n*+1. This method is effective if used correctly, as it more easily determines the number of stages. However, in some cases it is incorrectly calculated using *ℓ*=*n* instead of *ℓ*=*n*+1, resulting in incorrect stage number. As mentioned above, for the anions commonly used in DIB, *ℓ*=*n*+1 is often the case because Δ*d* has values in the range *d*_init_<Δ*d*<2*d*_init_, whereas *ℓ*=*n* for smaller ions with Δ*d*<*d*_init_, and *ℓ*=*n*+2 for very large ions with 2*d*_init_<Δ*d*<3*d*_init_. In practice, tetrafluoroborate shows Δ*d*=0.62~0.65 nm when solely intercalated, whereas co‐intercalation with solvents leads to a wide range of values between Δ*d*=0.71~1.07 nm depending on the solvents.[^33^, ^34^] Therefore, it is important to consider the size of the intercalate for appropriate calculation of the stage number. If Δ*d* is unknown, it is desirable to further examine other peaks, carefully consider index, and calculate the stage number, as described above.^[26]^

In addition, Raman spectroscopy can also be used to estimate the stage number. Graphite shows G band peak at around 1580 cm^−1^. With the intercalation of anions, a split peak appears on the slightly higher position than G band. In simple terms, the split peak appears because the graphene layer adjacent to the intercalate layer shows a peak at different positions from the graphene layer without adjacent intercalant layer. Therefore, the stage number can be estimated from the ratio of the intensity of the two peaks. For example, the peak intensity ratio for the stage‐4 compound is 1 : 1, while for stage‐3 it is 1 : 2, for stage‐2 and stage‐1 whole the original G band peak is observed at the higher position. Thus, more information on the stage number can be obtained by combining Raman spectroscopy measurements as well.

### 2.2 Chemical and electrochemical synthesis of acceptor‐type GICs

**Figure 3:**
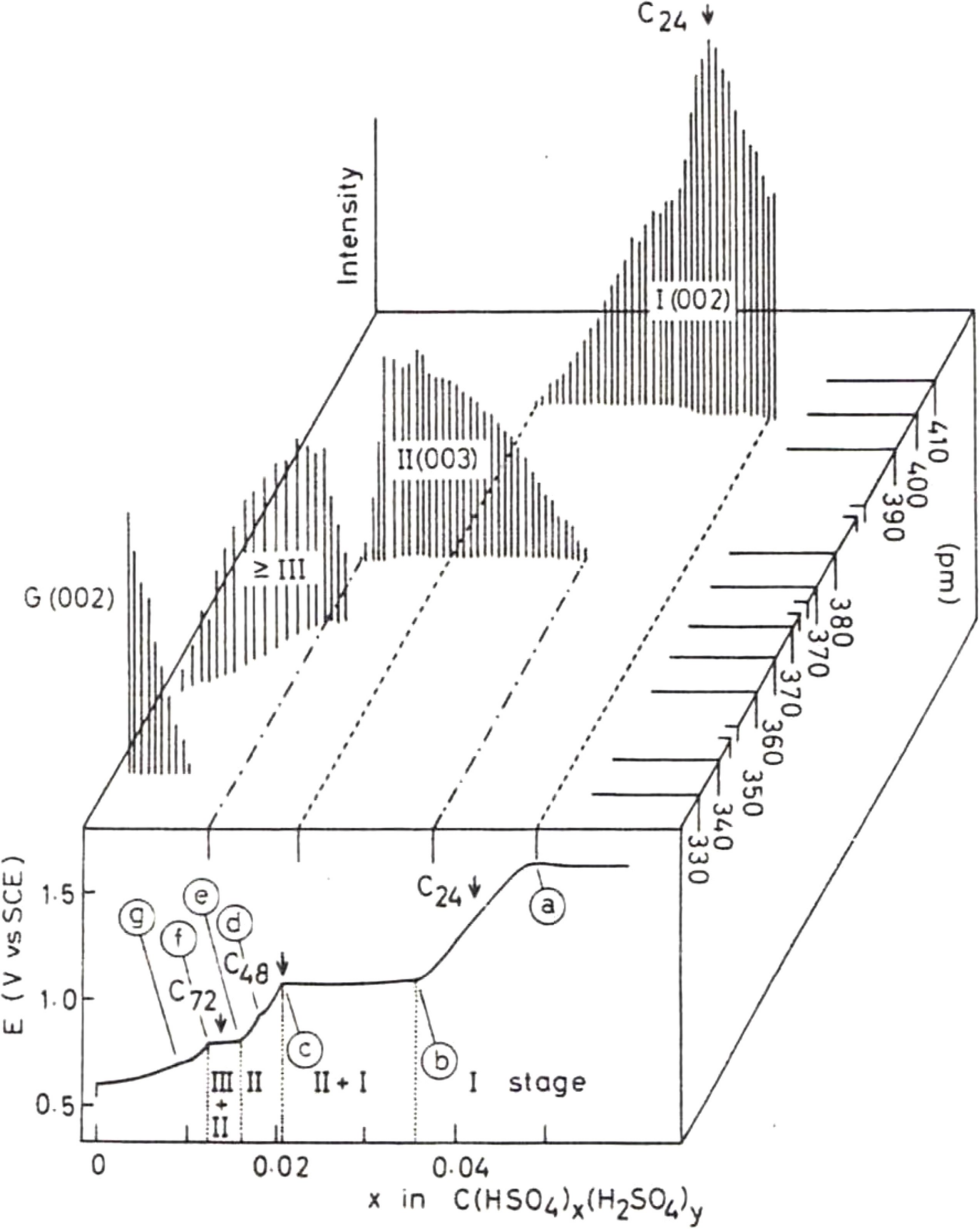
Variation of potential during constant current electrochemical oxidation of graphite electrode in a 96 % H_2_SO_4_ aqueous solution. The intensities of 00ℓ X‐ray diffraction peaks and their *d*‐spacings are also shown above. Reproduced with permission from Ref. [47]. Copyright 1983 Elsevier.

A great number of compounds have been reported thus far as intercalates of GICs. They are classified according to whether they form donor or acceptor compounds. When graphite is reduced, the resulting negative charge is compensated by cations inserted between the graphene layers, which provides donor type intercalation compounds. Donor type intercalates include alkaline metals, alkaline earth metals, transition metals, rare earth metals, tetra‐n‐alkylammonium cations, pyrrolidinium cations, etc. On the other hand, oxidation of graphite in the presence of anions can give acceptor type GICs. A large variety of acceptor type compounds have been also prepared and the intercalates include halogens,[^35^, ^36^, ^37^] metal halides,[^38^, ^39^] metal nitrates,^[40]^ oxoacids,^[41]^ etc. as shown in Table 1. Among these intercalates, fluorine and oxygen with large electronegativities and small sizes show quite different behavior in terms of chemical bonding, structure, electrical properties, etc. Hence, the characteristics of those unique GICs would be described later in another section. In this section, the synthesis method and structural features of the typical anion‐GICs are described. There are two major methods for synthesizing acceptor‐type GICs by oxidizing graphite: chemical and electrochemical methods. In case of chemical method, strong oxidizing reagents such as nitric acid, chlorine, fluorine gases, KMnO_4_, etc. are typically used. They are sometimes generated *in situ* from the intercalate; for example, metal chlorides decompose at elevated temperatures to form chlorine gas through the reaction 2MCl_x_→2MCl_x‐1_^−^+Cl_2_. The resulting chlorine oxidizes graphite and metal chlorides are intercalated. On the other hand, when graphite is electrochemically oxidized in appropriate electrolyte solutions, anions in them are intercalated to compensate the positive charge generated in graphite. One of the advantages of the electrochemical method is that it is possible to control the structure and composition of the resulting intercalation compounds by changing the applied potential or the degree of charging. Various anions such as oxoacid, metal halides, amides, etc. have been electrochemically intercalated into graphite. Since the oxidation potential of graphite is very high, electrolyte solutions with high oxidation stability are needed. Otherwise, decomposition of electrolyte occurs prior to the oxidation of graphite. Therefore, highly concentrated acid aqueous solutions with wide electrochemical windows have been used since the beginning of research of the electrochemical anion intercalation. More recently, organic electrolyte solutions, ionic liquids, or “water‐in‐salt” electrolytes are used especially for battery applications. The latter electrolytes have been extensively studied for DIB and are therefore discussed in chapter 4 on DIBs. The former electrolyte, which has a long history of research, is taken as an example here. Among the former, the electrochemical behavior of graphite in concentrated sulfuric acid aqueous solutions has been extensively studied so far.[^41^, ^42^, ^43^, ^44^, ^45^, ^46^, ^47^] Figure 3 shows the typical example of the variation of potential during constant current‐electrochemical oxidation of graphite (highly oriented pyrolytic graphite; HOPG) in 96 % sulfuric acid aqueous solution.^[47]^ The intensity of X‐ray diffraction peak and corresponding *d* spacing are also shown in Figure 3. A series of sharp and rounded kinks are observed, which are marked (a)‐(g). They were typically observed when graphite was oxidized to C_x_^+^ with x of (a): 21–20, (b): 29–27, (c): 48, (d): 56–54, (e): ca. 60, (f): 84–81 and (g): ca. 110, respectively. The (002) diffraction peak of graphite became weaker and the (00*ℓ*) diffraction peaks due to GIC appeared during electrochemical oxidation. The ascending and horizontal potential indicate single‐ and two‐phase GIC ranges as denoted by Greek numerals in Figure 3, respectively. They also performed dilatometry during a slow cyclic voltammetry at 1.4 mV min^−1^ and observed a small expansion of the sample at the kink (d). They concluded that this is ascribed to the closer packing of anions in the stage‐2 compound, which result in the slight increase in the interlayer spacing.

**Table 1:** Chemical species to form anions GICs.

Halogens	F, Cl, Br, ICI, IBr, etc.
Metal bromides	AlBr_3_, AuBr_3_, CdBr_3_, FeBr_2_, FeBr_3_, GaBr_3_, HgBr_2_, TlBr_3_, UBr_3_, etc.
Metal chlorides	FeCl_3_, AlCl_3_, NiCl_2_, CuCl_2_, MoCl_5_, SbCl_5_, NbCl_5_, etc.
Metal nitrates	Al(NO_3_)_3_, Ga(NO_3_)_3_, In(NO_3_)_3_, Co(NO_3_)_3_, Fe(NO_3_)_3_, Ti(NO_3_)_4_, etc.
Metal fluorides	SbF_5_, AsF_5_, VF_5_, NbF_5_, WF_6_, ReF_6_, OsF_6_, IrF_6_, PtF_6_, MoF_6_, UF_6_, etc.
Oxoacids	HNO_3_, H_2_SO_4_, HClO_4_, H_3_PO_4_, HSO_3_F, H_3_PO_4_, CF_3_COOH, HCOOH, PFOS (C_8_F_17_SO_3_H, etc.), etc.
Oxides	SO_3_, Cl_2_O_7_, N_2_O_5_, etc.
Fluorides	BF_3_, PF_5_, XeF_4_, XeF_6_, etc.

### 2.3 Composition of acceptor‐type GICs

**Figure 4:**
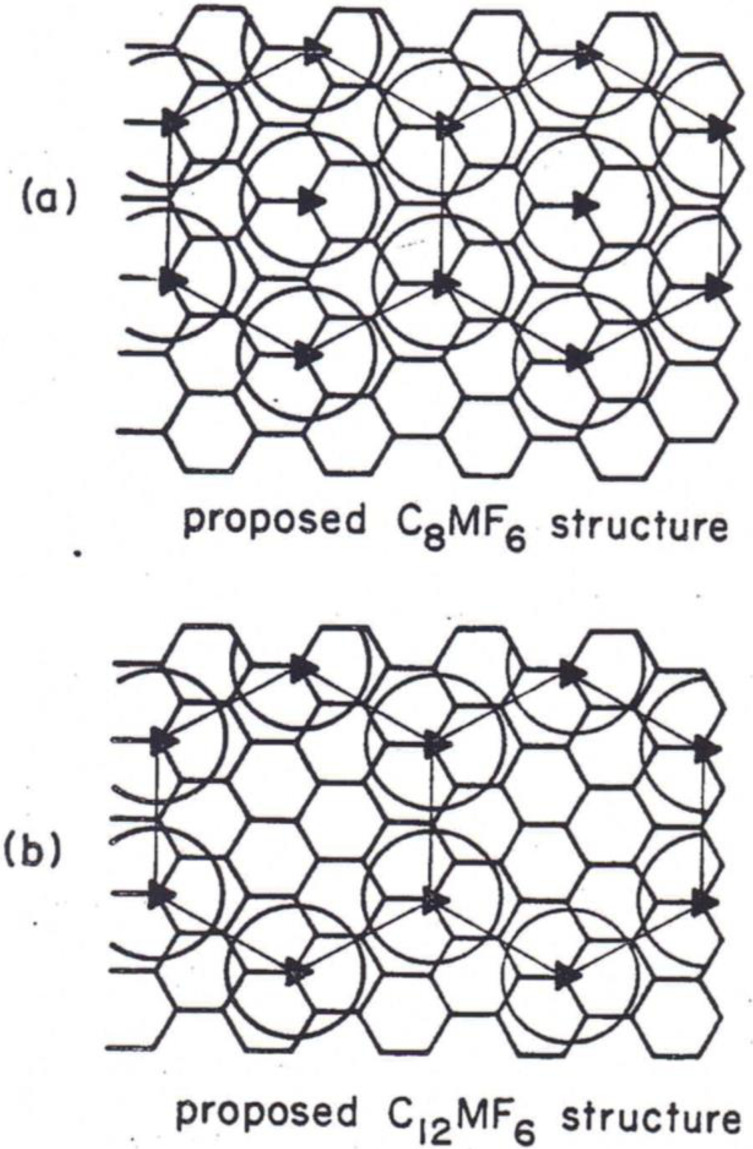
*In plane* structure models of C_8_MF_6_ and C_12_MF_6_. Reproduced with permission from Ref. [23]. Copyright 1982 Elsevier.

The capacity of graphite cathode is directly related to the composition of the resulting GIC, C_x_A where “A” indicates the intercalated anions and x is the ratio of carbon to anion. It has been reported that the saturated compositions of stage‐1 type alkaline or alkaline earth metals (denoted as M) intercalated graphite samples are MC_6_ or MC_8_. For example, in case of lithium and potassium, it is well‐known that they are LiC_6_ and KC_8_, therefore, the theoretical capacity of the graphite anodes of lithium‐ and potassium‐ion batteries are 372 and 279 mAh g^−1^, respectively. In case of anions, similar high contents of intercalated species are reported for those with polymeric structure in the interlayer space of graphite, such as transition metal chlorides (FeCl_3_ CoCl_2_, NiCl_2_, CuCl_2_, etc). The saturated composition reaches even C_4_A for these anions.^[48]^ The composition of C_8_A with a high content of anions was also reported for metal fluorides such as OsF_6_ and IrF_6_.[^23^, ^48^] For AsF_6_^−[49]^ and divalent fluoride of PtF_6_^2−^,[^23^, ^50^] the composition of C_12_A is reported. For these fluorides, *in‐plane* arrangements of intercalates on graphene layer are proposed as shown in Figure 4.^[23]^ Since these compositions are usually determined based on the weight change during reaction, it is not sure if all the intercalates are in the state of anion or not. However, these results indicate that there is enough space to accommodate such large amounts of intercalates in the interlayer space of graphite as observed for cations. In case of halogens, higher contents of intercalate have been also reported. Stage‐1 GICs of bromine and chlorine have not been prepared, however, the saturated compositions reached C_7‐8_Br[^51^, ^52^] and C_12_Cl for stage‐3,^[53]^ respectively. These mean that the *in‐plane* density of intercalates reach C_3.5‐4_Br and C_2.6_Cl, respectively. These high contents of intercalates are due to small sizes of halogens.

On the other hand, they are usually C_16‐24_A with lower anion contents, when they are electrochemically intercalated. For example, HSO_4_^−^ ions are electrochemically intercalated into graphite in a concentrated H_2_SO_4_ aqueous solution as already described and the composition of the resulting stage‐1 GIC was C_21‐24_HSO_4_ when it is determined based on the charge passed through the cell as shown above.^[47]^ Note that, in this case, 2.5 H_2_SO_4_ molecules per one HSO_4_^−^ are co‐intercalated, resulting in the composition of C_24_HSO_4_ ⋅ 2.5 H_2_SO_4_. Similar composition of C_24_ClO_4_ ⋅ 2.0 HClO_4_ was reported when it is prepared by the electrochemical oxidation in concentrated HClO_4_ aqueous solution.[^41^, ^42^] Therefore, the *in‐plane* density of the intercalates reaches C_6.9‐8_A if the neutral molecules are also included, which was comparable to those observed for chemically prepared anion GICs.

**Figure 5:**
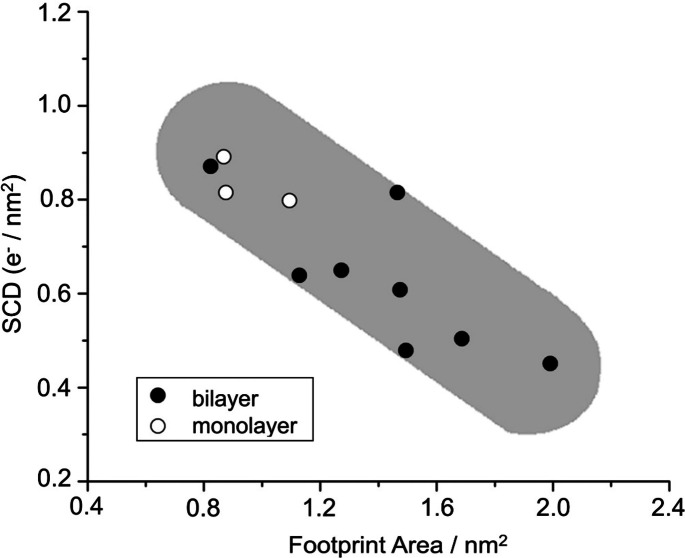
Graphene sheet charge densities (SCD) vs footprint areas for stage‐1 *N,N*‐pyrrolidinium GICs. Reproduced with permission from Ref. [54]. Copyright 2016 American Chemica Society.

It would be important to mention the effect of the size of intercalate on the structure and composition of GIC. Lerner et al. investigated it for various cationic intercalates and found that the linear relationship between the charge density of graphene sheet and footprint area, as shown in Figure 5.^[54]^ Here, the footprint area is the occupied one of a cation located on the graphene sheet. This indicates that the content of the anion with a larger footprint area would be also smaller.

## 3 Covalent‐type GICs: the case of oxygen and fluorine intercalation

As mentioned above, the GICs obtained by intercalation of oxygen and fluorine significantly differ from other GICs in terms of structural and chemical characteristics. This is because, unlike other ions, they form covalent bonding with carbon atoms inside graphite. For oxygen, electrochemical intercalation of O^2−^ ions has not been reported yet and only the formation of OH^−^ GIC has been suggested.^[55]^ Instead, introduction of oxygen into graphite results in formation of graphite oxide or graphene oxide, both abbreviated as GO. In particular, graphene oxide has been intensively studied especially after the “discovery” of graphene in 2004^[56]^ because it is easily exfoliated in solvents and reduction of it has been expected to provide graphene by removing oxygen functional groups from it.[^57^, ^58^, ^59^, ^60^, ^61^, ^62^, ^63^] In addition, reduced GO (rGO) and its related materials have recently become strong candidates as cathode materials for DIBs, as described in the next chapter. On the other hand, GICs obtained by introducing fluorine have been used as cathode materials for lithium primary batteries.^[64]^ Recently, fluorine‐GIC has also shown promise as cathode materials for fluoride shuttle batteries,^[65]^ which is a type of DIB in the broad sense. Therefore, this chapter outlines the synthesis methods and structural and chemical characteristics of these materials.

### 3.1 Synthesis and structure characterization of GO

**Figure 6:**
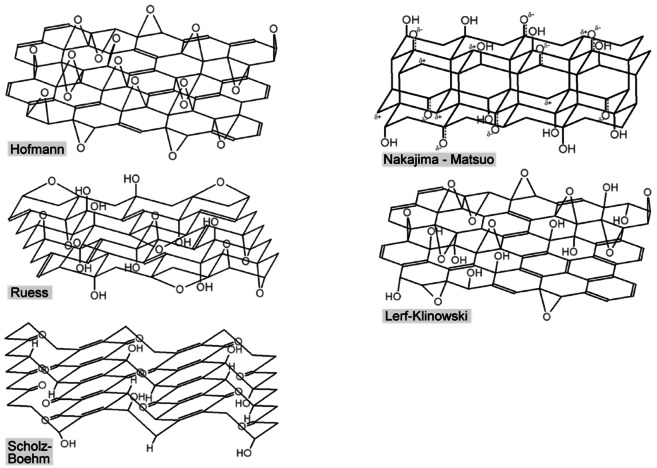
Structure models of graphite oxide. Reproduced with permission from Ref. [66]. Copyright 2016 American Chemica Society.

**Figure 7:**
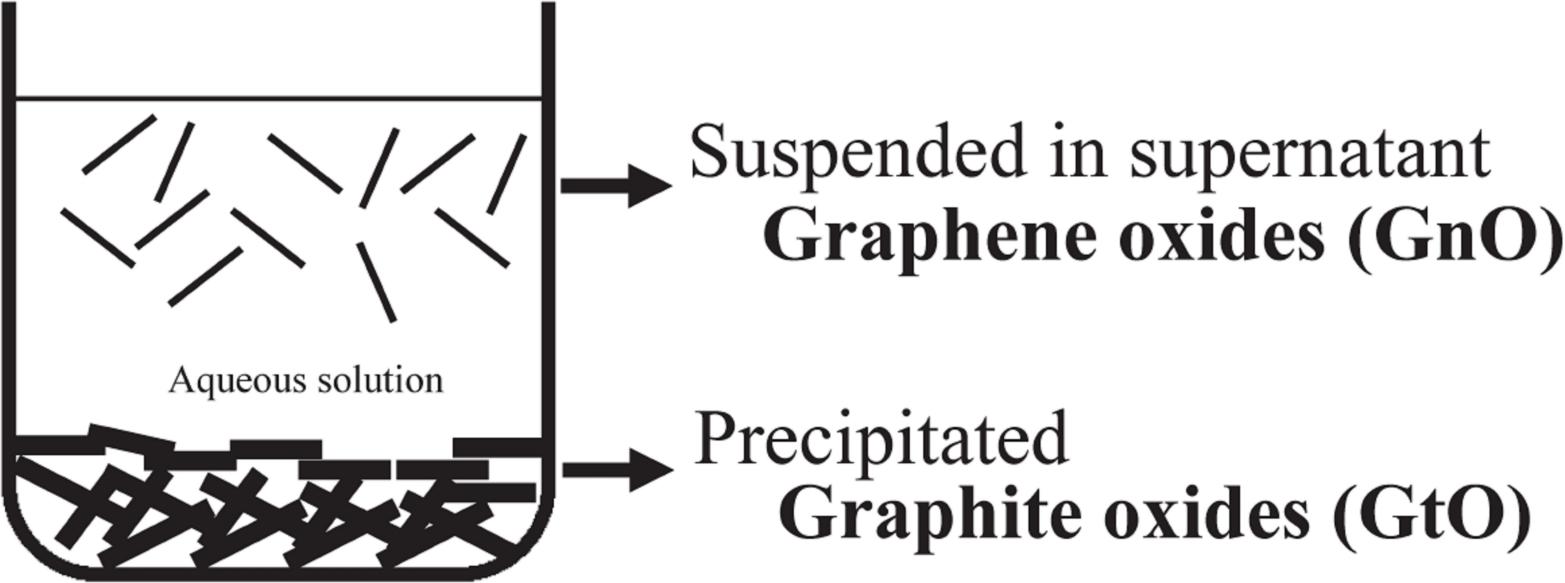
The differentiation between graphite oxide and graphene oxide, together with their abbreviated expressions. Reproduced from Ref. [81]. Copyright 2022 Copyright The author(s).

GO contains covalently bonded oxygen atoms in the form of hydroxyl, ether, carboxyl, etc. Several structure models were proposed previously as summarized in Figure 6.^[66]^ Though they have still been modified, Lerf–Klinowski model[^67^, ^68^] seems to be widely accepted. The interlayer spacing and composition of it greatly vary depending on the conditions such as starting graphite, oxidation method, ambient humidity, etc. The widely used synthetic method of GO is the chemical ones.[^69^, ^70^, ^71^, ^72^] This method involves treating the raw graphite with strong oxidizing reagents or concentrated acids and has a very long history. However, this method inevitably generates large quantities of waste. Therefore, electrochemical synthesis of GO has gained attention as an alternative method with a lower environmental impact.[^73^, ^74^, ^75^, ^76^] The electrochemical method employs concentrated oxoacid aqueous solutions as electrolyte solution. This method is commonly used for the exfoliation of graphite to obtain graphene or few layer graphene as summarized in several review papers.[^77^, ^78^, ^79^, ^80^] In case of the preparation of graphene, it is preferable to minimize the introduction of oxygen atoms or defects into the resulting materials. However, many papers seem to call the materials electrochemically exfoliated and dispersed in the electrolyte solution as GO, even though the oxygen contents of them are low. In addition, rGO, obtained by the reduction of GO, has potential applications in electrochemical devices. However, it exhibits capacitor‐like electrochemical behavior due to its high specific surface area with little internal space between the layers. In order to focus on GO as a precursor for DIB cathode active materials, in this review, we focus here on the materials which contain a large amount of oxygen and provide no X‐ray diffraction peak due to residual graphite, namely “graphite oxide”. Here, we abbreviate it as GtO according to the recent proposal by Inagaki and Takai.^[81]^ They proposed that thin flakes suspended in the supernatant of acidic solution just after synthesis can be called “graphene oxides”; GnO and the precipitate “graphite oxides”; GtO as schematically illustrated in Figure 7.

#### 3.1.1 Overoxidation of anion‐GIC and formation of GtO

**Figure 8:**
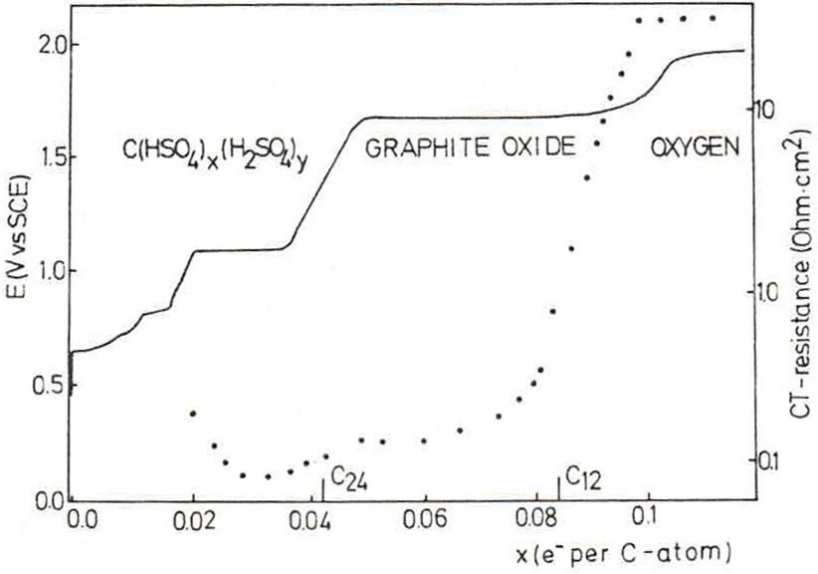
Variation of potential and charge transfer resistance measured during constant current oxidation of HOPG in a 96 % H_2_SO_4_ aqueous solution. Reproduced with permission from Ref. [47]. Copyright 1983 Elsevier.

**Figure 9:**
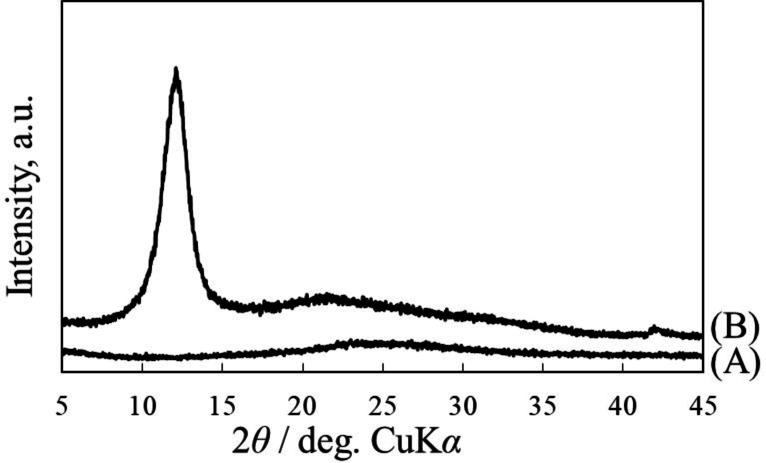
X‐ray diffraction patterns of graphite sheet (A) just after constant current electrochemical oxidation in 11.6 M HClO_4_ aqueous solution and (B) then immersed in water overnight. The electrochemical oxidation was performed under the similar condition reported in Ref. [87].

When stage‐1 GIC was further oxidized (overoxidized), a potential plateau at 1.6 V is additionally observed, as shown in Figure 8.^[47]^ Impedance measurement indicated that charge transfer resistance drastically increased after graphite was charged to C_12_^+^ as shown in Figure 8 with dotted line. Therefore, it was considered that GtO was formed at this plateau,[^47^, ^82^, ^83^, ^84^, ^85^, ^86^] though no direct evidence such as X‐ray diffraction datum was provided. Then, the potential further increased and reached a plateau of oxygen evolution. The onset of oxygen evolution greatly dependent of the crystalline size of graphite. This behavior is commonly observed in concentrated oxoacids such as perchloric acid, nitric acid, etc. Nakajima and Matsuo reported that the onset of oxygen evolution was C_2_^+^ when graphite sheet was oxidized in 11.6 mol dm^−3^ perchloric acid solution.^[87]^ The overoxidation of stage‐1 perchloric acid‐GIC resulted in the amorphous material just after electrochemical oxidation. However, after the immersion of it in methanol overnight, the formation of GtO was confirmed by X‐ray diffraction measurement as shown in Figure 9. GtO was also formed by the overoxidation of stage‐2 GIC in a 9.2 mol dm^−3^ solution, however, that of stage‐3 GIC formed in 4.6 mol dm^−3^ solution did not provide GtO.

**Scheme 1:**
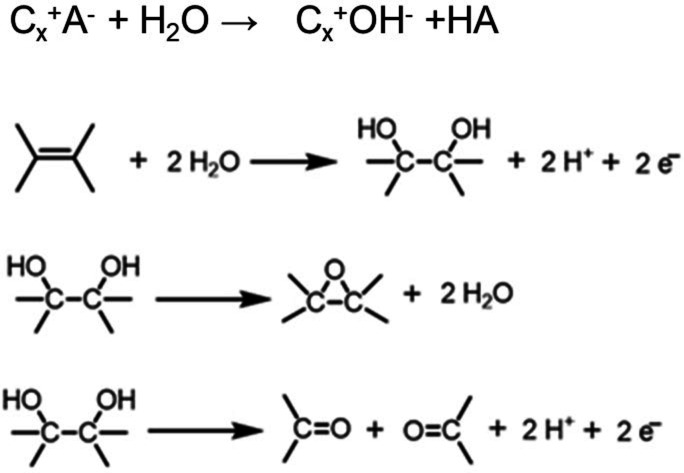
Formation process of graphite oxide proposed in Ref. [84].

Besenhard et al.^[88]^ and later Beck et al.^[84]^ suggested that the formation of OH^−^‐GIC as the result of the nucleophilic reaction of anion‐GIC with water occurred during overoxidation of anion‐GIC, based on the decrease in the interlayer spacing of it. At the same time, electrochemical oxidation of C=C and addition of water molecules provide vicinal OH groups and then, epoxy rings may be formed through chemical dehydration process. Further electrochemical oxidation of vicinal OH groups leads to the C−C cleavage to form carbonyl groups. These are schematically illustrated in Scheme 1. It has been also pointed out that oxygen containing radicals oxidize anion‐GIC to form GtO, as shown later.

The other interesting phenomenon during galvanostatic overoxidation of anion‐GICs is potential oscillations with periods of up to several hours.[^78^, ^79^, ^80^, ^81^, ^89^] It has been reported that the interlayer spacing changes during oscillation and it is maximum and minimum at the potential minimum and maximum, respectively, based on the X‐ray diffraction measurements.^[82]^ Beck et al. proposed zone model of the formation of C‐OH group with hydrogen bonds and their conversion to C=O groups in the intercalate layers for this potential oscillation.^[84]^

#### 3.1.2 Choice of electrolyte solution

**Figure 10:**
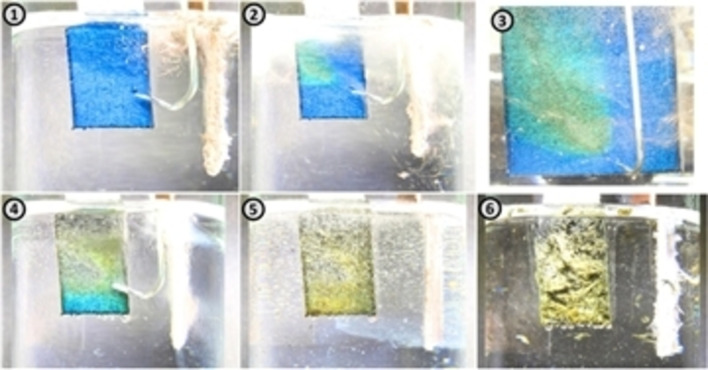
Oxidation of graphite by targeted spraying of the blue GIC with water. Image 1: Blue graphite intercalation compound. Image 2 and 3: Spraying the GIC with water. Image 4 and 5: The gradual mixing of the organic electrolyte with water oxidizes the entire graphite surface. Image 6: After 5 minutes of electrolysis. Reproduced from Ref. [95]. Copyright 2019, The Author(s).

As electrolyte solutions, aqueous concentrated acids such as H_2_SO_4_ and HClO_4_ have been usually used and as described above, electrolyte concentration is one of the important factors to obtain GtO. Lowe et al. investigated the effect of the concentration of the electrolyte during constant current oxidation of graphite in more detail and revealed that GtO formed even in 2 mol dm^−3^ H_2_SO_4_, however, complete oxidation of graphite was achieved in higher concentrations than 11.6 mol dm^−3^.^[90]^ When high voltage or large current are applied, the concentration of the solution is not necessarily high. Sahoo et al. found that in case of HNO_3_ and HClO_4_ aqueous solution, GtO was obtained even in 1 mol dm^−3^ solution, though the yield was low. GtO was successfully formed even in 0.2 mol dm^−3^ sodium citrate aqueous^[91]^ or 0.1–1.0 mol dm^−3^ (NH_4_)_2_SO_4_ solutions.[^92^, ^93^] It is possible to use HBF_4_ aqueous or methanol solutions as reported by Campéon et al.^[94]^ Comparing with the oxidation in a H_2_SO_4_ aqueous solution, exfoliation and destruction of graphite framework was considerably suppressed. They also found that the addition of methanol prevented the gas generation and destruction of graphite, enabling complete oxidation of it. Electrochemical oxidation of graphite foil in organic solvent has been also reported. Schedy et al. used 40 mL of 1 mol dm^−3^ NaClO_4_‐propylene carbonate/dimethyl carbonate containing 2 mL of water as an electrolyte. By applying 10 V, the color of graphite foil changed blue and then turned to yellow, which indicated the formation of ClO_4_^−^‐GIC and GtO, respectively.^[95]^ They have shown that the color change from blue to yellow was observed only when water molecules were deliberately brought to the surface of electrode using a syringe as shown in Figure 10. They suggested that water molecules were co‐intercalated into graphite with ClO_4_^−^ ions and then decomposed to form oxygen containing radical intermediates to react with GIC, providing GtO.

#### 3.1.3 Effect of potential

Gurzęda et al. reported the effect of potential during the electrochemical oxidation of graphite flake in 8 mol dm^−3^ HClO_4_ solution by linear sweep voltammetry at a low rate of 0.01 mV s^−1^.^[96]^ For complete oxidation of graphite, 1.4 V vs Hg/Hg_2_SO_4_ (0.615 V vs SHE) was needed. This value corresponds to 2.0 V vs SHE and similar to the plateau at 1.85 V observed in Figure 8. On the other hand, in order to achieve complete oxidation of graphite in the electrolyte solutions of lower concentrations, higher voltages are needed. For example, in 1 mol dm^−3^ NaOH aqueous solution, 3–10 V were needed.^[97]^ Application of high voltage can provide GtO even in non‐acidic solutions such as 0.5 mol dm^−3^ (NH_4_)_2_SO_4_ in a short oxidation time.^[93]^

#### 3.1.4 Oxidation methods

Two‐step method was proposed by Cao et al.^[98]^ They galvanostatically prepared stage‐1 GIC in concentrated H_2_SO_4_ aqueous solution in the first step, then constant voltage of 10 V was applied to it in 0.1 mol dm^−3^ (NH_4_)_2_SO_4_ solution in the 2nd step. Paste like product was formed after washing and GtO was obtained by freeze drying it. When graphite foil with 2.5 cm wide, 10 cm long and 0.5 mm thick was used, they obtained 10.5 g of GO. By using 50 % H_2_SO_4_ solution and applying 5 V during the 2nd step, GtO was obtained in a very short time of less than 30 min.^[99]^ They investigated the composition of gaseous products during the electrochemical oxidation in 50 % H_2_SO_4_ at 5 V and found that anion‐GIC efficiently inhibited the evolution of O_2_ gas at the anode. This indicated that the radical intermediates such as *OH, *O and *OOH were adsorbed in GIC, which facilitated the formation of GtO at a very high rate. Chen et al. have proposed the photosynergetic electrochemical synthesis of GtO in 0.1 mol dm^−3^ oxalate acid and 0.05 mol dm^−3^ Na_2_SO_4_,.^[100]^ Graphite rod was first treated using a square‐wave potential (high potential: 10 V, low potential: −0.5 V, period: 2 s) for 20 min and then electrolyzed with a constant potential of 15 V. During electrolysis, the system was illuminated with a xenon lamp light source to generate radicals such as *OH, etc.

#### 3.1.5 Type of graphite

**Figure 11:**
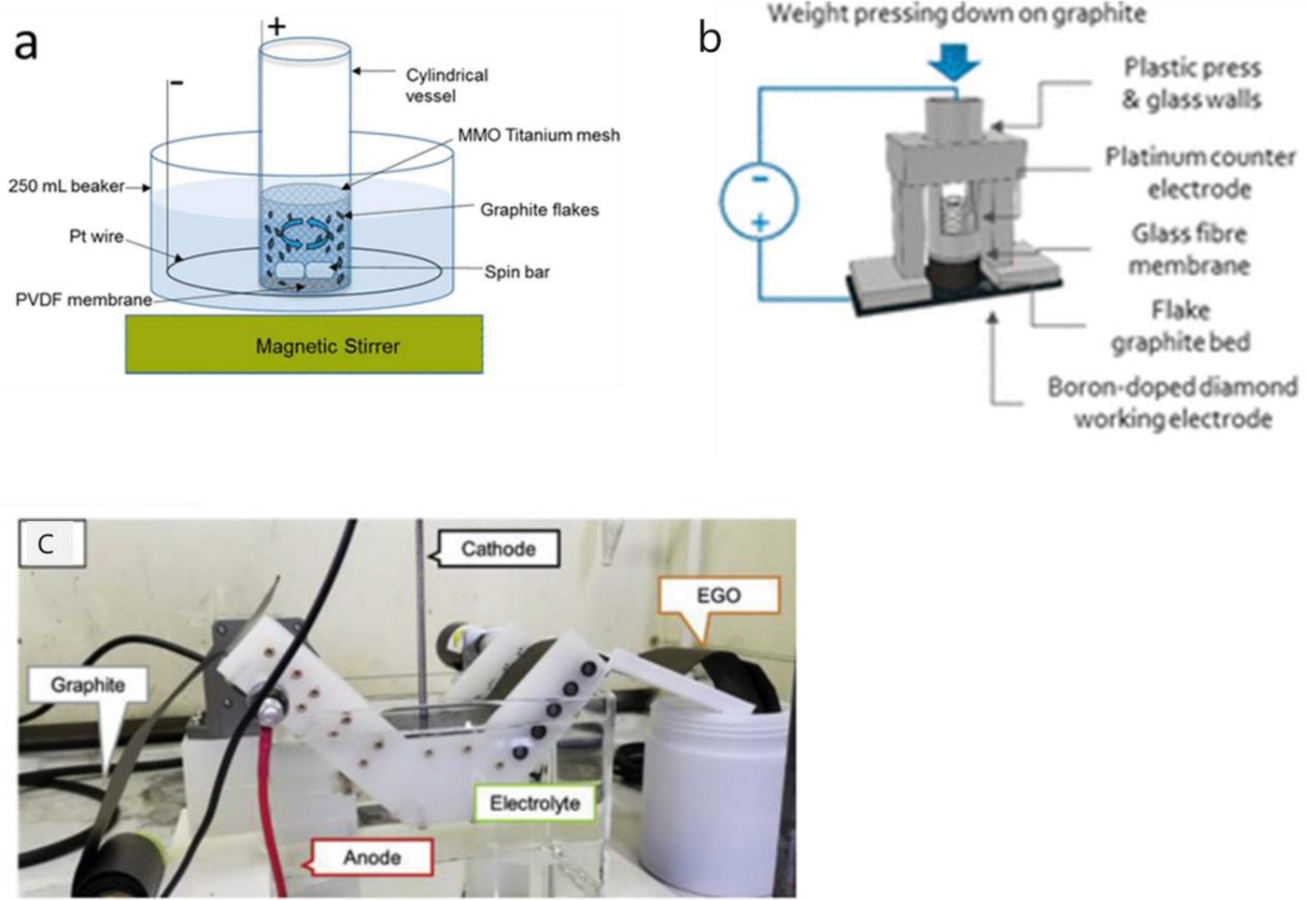
Electrochemical apparatus for producing graphite oxide. (a) Schematic drawing of the mechanically assisted electrochemical exfoliation setup (b) illustration of the small‐scale tubular reactor used for fundamental studies of graphite oxidation and (c) continuous flow electrochemical treatment system developed using graphite sheet to produce GO. (a) Reproduced with permission from Ref. [110]. Copyright 2016 American Chemical Society. (b) Reproduced with permission from Ref. [111]. Copyright 2019 American Chemical Society. (c) Reproduced with permission from Ref. [94]. Copyright 2020 Elsevier.

The other issue for the mass production of GtO is the type of graphite electrode. Monolithic graphite electrodes such as highly oriented pyrolytic graphite (HOPG), graphite sheet,[^99^, ^101^] disc[^102^, ^103^] and rod[^104^, ^105^] are usually used to prepare GtO. This is because graphite electrode is greatly expanded during electrochemical oxidation and even removed from the electrode, which leads to incomplete oxidation of it or low yield of GtO. Therefore, in case of the electrochemical oxidation of graphite flakes which is more favorable for mass production of GtO, it is necessary to consider how to maintain the electrical contact between graphite flakes (in most cases >100 μm) and as well as between them and current collector during oxidation. Metal mesh such as Pt or boron‐doped diamond (BDD) are used to support the graphite flakes,[^96^, ^106^, ^107^, ^108^, ^109^] however, in order to prepare a large amounts of GtO, improvement of experimental apparatus is needed. Yu et al. succeeded to prepare GtO flakes at high yields of upto 38.8 % by mechanical stirring of the electrolyte solution containing graphite flake packed in an anode compartment with PVDF membrane using the apparatus shown in Figure 11(a), though X‐ray diffraction peak due to residual graphite was still observed.^[110]^ Lowe et al. pressed graphite flakes on to the current collector to support them as shown in Figure 11(b).^[111]^ They prepared GtO in various concentration of aqueous sulfuric acid solutions by applying different currents as described in section 3.1.2. They obtained GtO showing negligible X‐ray diffraction peak due to residual graphite when boron‐dope diamond as a current collector. The resulting GtO was well dispersed in various solvents and contained less defects when compared with chemically prepared GtO. Campéon et al. have developed mass production of GtO using graphite sheet with continuous flow electrochemical treatment system as shown in Figure 11(c).^[94]^ Graphite sheet was gradually fed in the electrolyte solution, and they succeeded to prepare 32 g of GtO in one day.

### 3.2 Overoxidation of fluorine GIC

**Figure 12:**
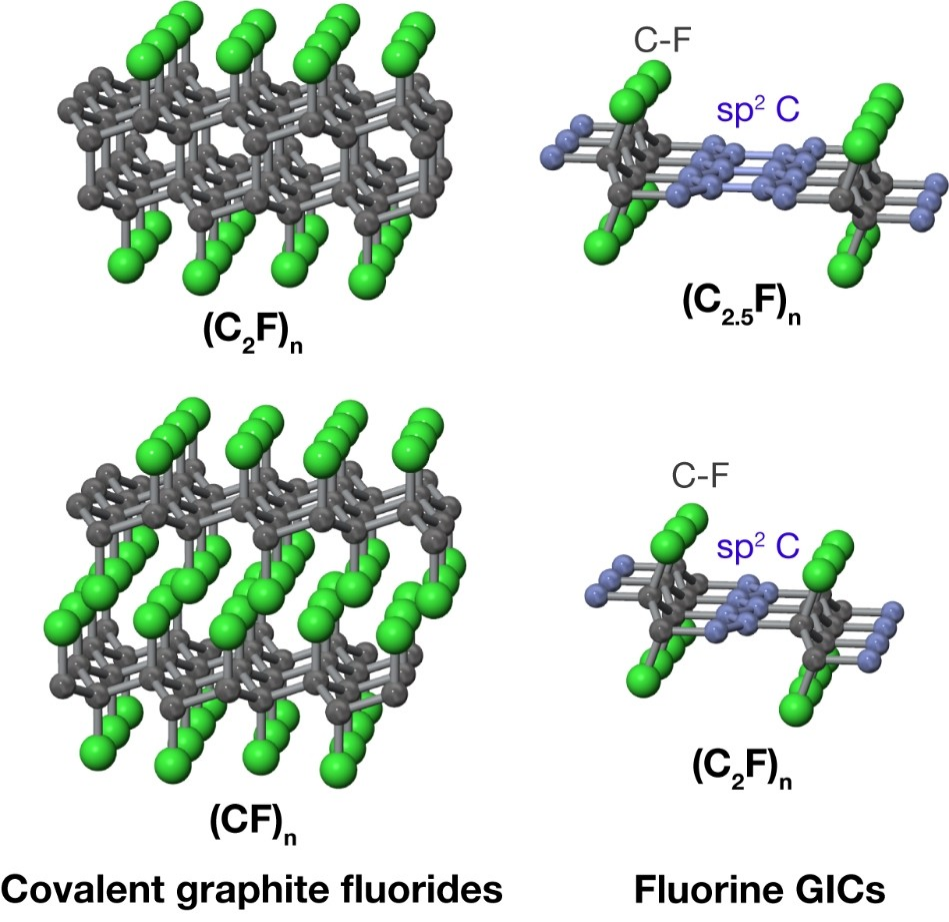
Structure models of covalent type fluorine‐intercalated graphites, (CF)_n_, (C_2_F)_n_ and stage‐1 C_x_F. Reproduced with permission from Ref. [121]. Copyright 2020 Elsevier.

When graphite is reacted with molecular fluorine at elevated temperatures, fluorine atoms are directly bonded to carbon atoms via covalent bonding, forming covalent type graphite intercalation compounds, polycarbon monofluoride, (CF)_n_ or poly(dicarbon monofluoride), (C_2_F)_n_.[^37^, ^112^, ^113^] In these materials, sp^2^ type carbon atoms of graphite are converted to sp^3^ type ones, and therefore they become insulator. At lower temperatures and in the presence of catalyst, non‐stoichiometric acceptor type fluorine GICs, C_x_F (x indicates carbon/fluorine ratio) are formed.^[113]^ For stage‐2 or higher GICs, fluorine atoms are ionically bonded, and the stoichiometry of C_5.4_F (*in‐plane* density of C_2.7_F) is proposed.^[114]^ Stage‐1 intermediate materials are also obtained in which two regions consisting of sp^3^ carbons covalently bonded to fluorine and aromatic sp^2^ carbons co‐exist. The composition reaches C_1.2_F, when it was prepared using KAgF_4_ as a catalyst.^[115]^ The carbon‐fluorine bonding in these materials is previously called semi‐ionic or semi‐covalent one, based on the intermediate C1s and F1s binding energies observed by XPS.[^116^, ^117^] However, neutron diffraction and NMR studies revealed that it is essentially covalent and the intermediate XPS binding energies are ascribed to the hyperconjugation of C−F bonds with neighboring sp^2^ regions.[^118^, ^119^, ^120^, ^121^] The structure of these fluorine intercalated graphite with different structure are shown in Figure 12.

Electrochemical intercalation of fluorine has been also reported in hydrofluoric acid solution[^122^, ^123^, ^124^] or molten salt of KF ⋅ 2HF.[^125^, ^126^, ^127^] Only high stage GIC was obtained in the latter probably due to the high operating temperature above 100 °C, considering that the entropy greatly decreases during intercalation reaction. In hydrofluoric acid, stage‐2 C_12_HF_2_ was obtained by Lerner et al. Later, Matsuo et al. found that by increasing the current density during the constant current electrochemical oxidation of graphite sheet, it is possible to increase the potential of the electrode due to the overpotential needed for decomposition of the electrolyte.[^123^, ^124^] By increasing the current density, the potential of graphite electrode can reach that oxidizing graphite at higher levels. They obtained stage‐1 fluorine GIC, and the composition reached C_2.8_F.^[123]^ At higher current densities, even the introduction of oxygen into C_x_F occurred.^[124]^ Homogeneous distribution of oxygen and fluorine in the resulting material was confirmed by the ^13^C NMR measurement.

## 4 Application of graphite as the cathode of dual‐ion batteries

The electrochemical intercalation/de‐intercalation of anions into/from graphite are utilized as the charging/discharging reactions of cathode in DIBs, respectively. Among the performance of graphite cathode, the capacity of it is related to the composition of the resulting GIC and should be unique value for the intercalated anions when it is operated in appropriate electrolyte solutions. Here, the charge‐discharge behaviors of graphite cathode in various electrolyte solutions are first mentioned. On the other hand, the rate capability and cycling properties of graphite cathode is greatly dependent on its size, the structure of composite electrode, thickness of the electrode, etc. Although there are many papers reporting excellent performance, it is rather difficult to compare them with each other. Therefore, here we focus on the fundamental properties such as interfacial phenomenon and kinetics of anion intercalation. In addition, many types of carbon materials other than graphite have also been tested for the cathode of DIBs and some of them show superior performance. Here, we mention the carbon materials which mainly store anions in their interlayer spacing as is the case of graphite, and do not cover those that use the charge capacity of the electric double layer, such as capacitors.

### 4.1 Charge‐discharge behaviors of graphite in various electrolyte solutions

**Figure 13:**
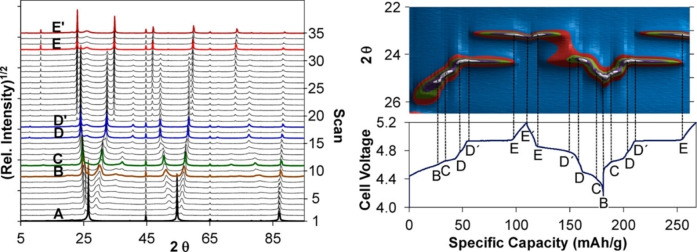
*In situ* XRD of graphite during charge and discharging in topographical format with corresponding voltage‐capacity curves. (Relative Intensity)^1/2^ is represented by colors in the legend: (white) 500–400 (purple) 400–300 (green) 300–200 (red) 200–100 (blue) 100–0. Reproduced with permission from Ref. [27]. Copyright 2015 American Chemical Society.

As briefly mentioned in chapter 2, organic electrolyte solutions, ionic liquids and “water‐in‐salt” electrolytes have been widely considered as electrolytes for DIBs. In particular, organic electrolytes have been the most extensively studied since the dawn of DIB. In 2000, Seel and Dahn reported electrochemical intercalation/de‐intercalation of PF_6_^−^ into/from graphite, and reversible stage transformation has been shown during charge and discharge cycles.^[128]^ They have shown that the capacity of graphite cathode reached approximately 100 mAh g^−1^ (C_22.3_PF_6_) or even 140 mAh g^−1^ (C_15.9_PF_6_), when it is charged up to 5.5 V using an appropriate electrolyte. Later, Read has investigated the reaction of graphite cathode during charge‐discharge cycle in detail by *in situ* X‐ray diffraction and dilatometry as shown in Figure 13.^[27]^ The potential profile during charging in 3 mol dm^−3^ LiPF_6_/ethyl methyl carbonate (EMC) was like that in concentrated H_2_SO_4_ aqueous solution shown in Figure 3 and both ascending and horizontal potentials were observed during charging. He revealed that the formation and transition of the GIC phases in the following manner, stage‐4 C_96_PF_6_, stage‐3 C_72_PF_6_, stage‐2 C_48_PF_6_, stage‐1 C_24_PF_6_ and finally stage‐1 C_20_PF_6_. Therefore, the capacity reached 112 mAh g^−1^. The expansion of the electrode reached 130 % at the end of charge, as expected from the size of intercalated PF_6_^−^ ions of 0.43 nm. When the other anions are intercalated, similar potential profiles to that for PF_6_^−^ are also observed, though kinks or plateaus are observed at different oxidation levels or potentials, respectively, depending on the electrolyte solutions. Among them, the maximum stoichiometry of TFSA^−^ intercalated graphite varied depending on the temperature, and C_20_(TFSA) (111.3 mAh g^−1^) was reported.^[129]^

**Figure 14:**
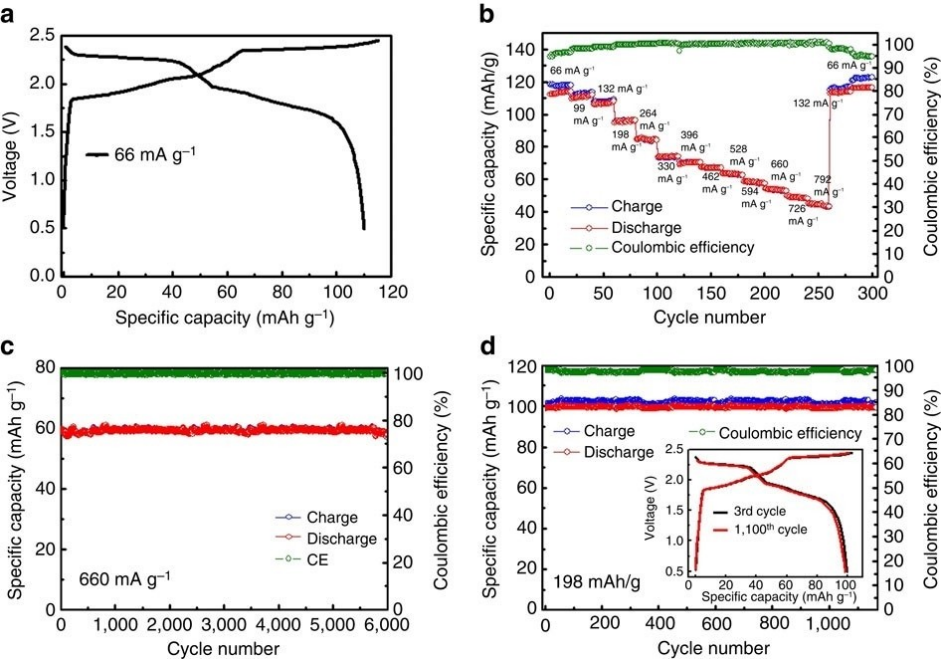
(a) Galvanostatic charge‐discharge curves of an Al/NG cell at a current density of 66 mA g^−1^. (b) Capacity retention of an Al/NG cell cycled at various current densities. (c, d) Long‐term stability test of an Al/NG cell at 660 and 198 mA g^−1^, respectively. Reproduced from Ref. [131]. Copyright 2017, The Author(s).

**Figure 15:**
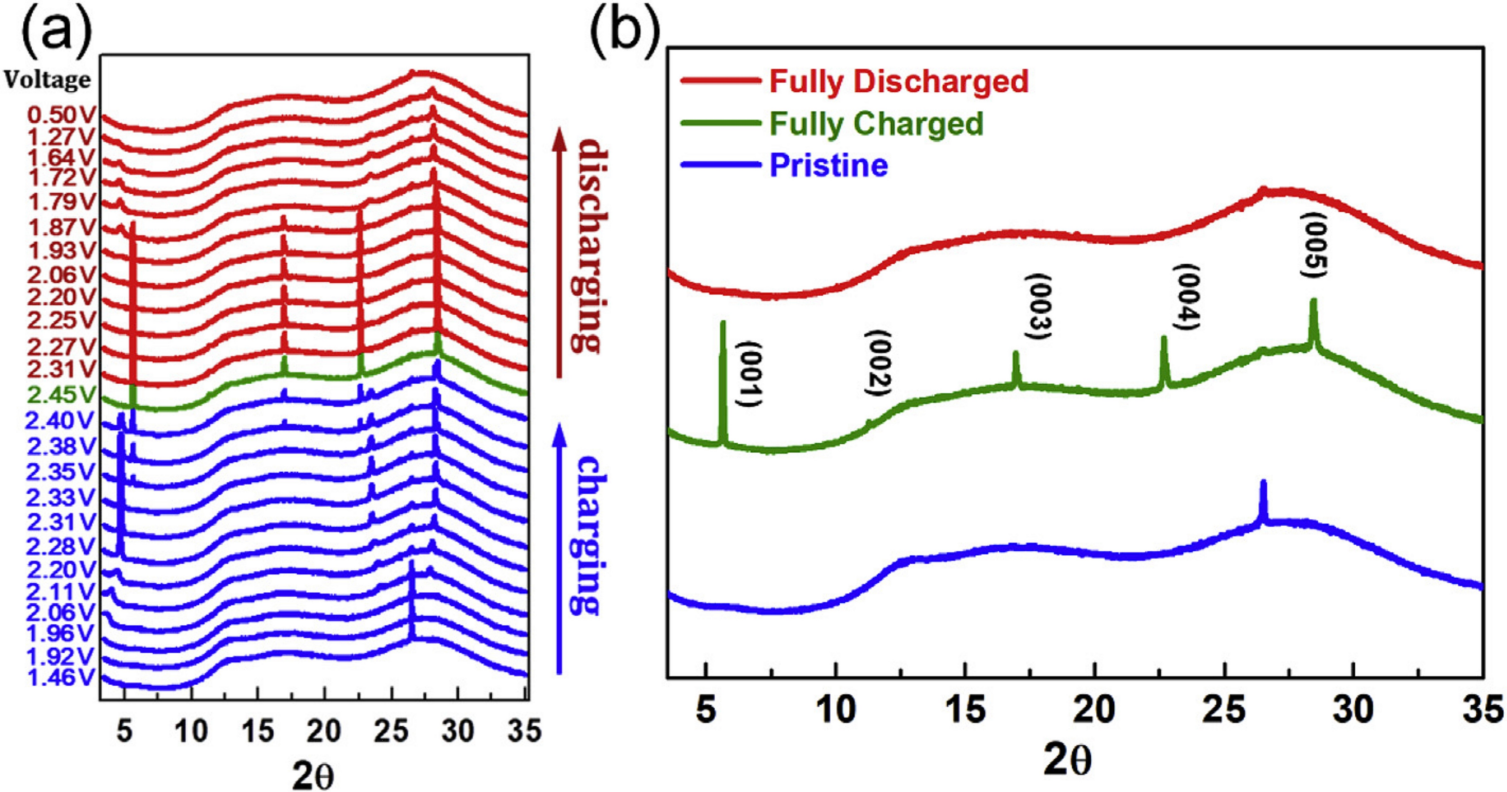
(a) The *in situ* XRD patterns of NG/Al battery collected during second cycle at 100 mA cm^−2^ which charging and discharging curves is shown at the right. (b) The selected XRD pattern of NG/Al battery at pristine, fully charged and fully discharged status. Reproduced with permission from Ref. [132]. Copyright 2019 Elsevier.

**Figure 16:**
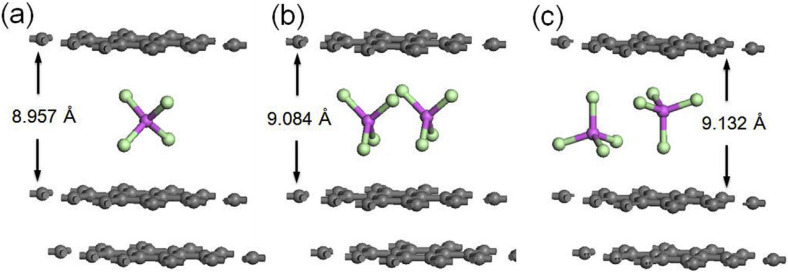
Optimized structure of (a) X‐shaped loosed model (b) X‐shaped packed model and (c) upside‐down model includes one or two AlCl_4_^−^ ions intercalation in the graphite layer which are constructed by 3×3 repetition of the graphite primitive unit cell. Reproduced with permission from Ref. [132]. Copyright 2019 Elsevier.

The other well studied system is the intercalation of AlCl_4_^−^ ions.[^130^, ^131^, ^132^, ^133^, ^134^, ^135^, ^136^] Dai and co‐workers proposed the use of graphite cathode for aluminum ion battery in an ionic liquid of [EMIm]Cl (1‐ethyl‐3‐methylimidazolium chloride) containing AlCl_3_.^[131]^ They observed the diffraction peaks at 2*θ*=28.25° (*d*=0.315 nm) and 23.56° (*d*=0.377 nm) for fully charged sample (62 mAh g^−1^, C_36_^+^) and indicated the formation of stage‐4 GIC as shown in Figure 14. Later, they succeeded to increase the capacity to 110 mAh g^−1^, C_20_^+^ by using natural graphite flakes. The X‐ray diffraction data suggested the formation of stage‐3 GIC at the end of charging. Wang et al. reported the intercalation mechanism of this battery using operand synchrotron X‐ray technique.^[132]^ As shown in Figure 15, a series of (00*ℓ*) diffraction peaks from (001) to (005) were clearly observed at the end of charge of 2.45 V. This means that stage‐3 GIC with an interlayer spacing of 1.592 nm formed. They also measured X‐ray absorption and concluded that tetrahedral AlCl_4_^−^ ions are intercalated and the change in the spectral feature during charging suggested the X shape closely packed model as shown in Figure 16 is probable. This could be the reason for the high density of AlCl_4_^−^ in the interlayer space of graphite.

Recently, electrochemical anion intercalation has been widely studied in aqueous electrolytes for application to DIBs. The problem with ordinary aqueous electrolyte solutions is the narrow potential window of water. To overcome this, it is necessary to kinetically and/or thermodynamically suppress the oxidative decomposition of water. As mentioned in section 3.2, one method to kinetically suppress this is to use overpotential. It is utilized to obtain [ZnCl_4_]^2−^‐GIC in an aqueous ZnCl_2_ solution in which decomposition of water occurs at lower potentials than that of the oxidation of graphite.^[137]^

**Figure 17:**
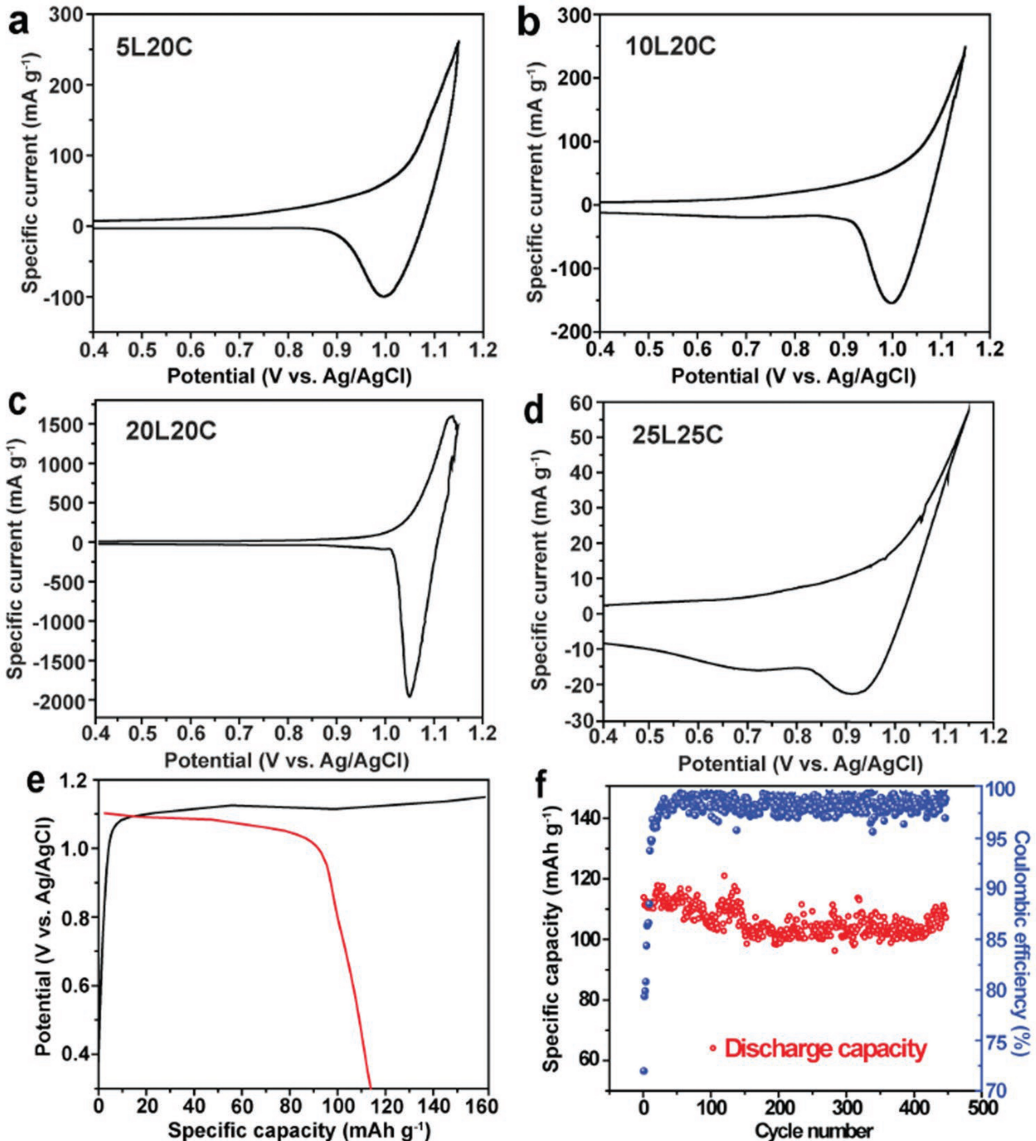
Electrochemical characterization of graphite electrode in different electrolytes. (a–d) CV curves at a scan rate of 0.1 mV s^−1^, (e) 1st charge‐discharge profiles and cycling performance of graphite in 20 L20 C at 50 mA g^−1^. Reproduced with permission from Ref. [138]. Copyright 2022 Wiley.

Alternatively, the use of highly concentrated electrolyte solutions is a method of thermodynamically inhibiting the oxidative decomposition of water. In particular, those with extremely high concentrations where the weight and volume of salt is larger than that of solvent water are called “water‐in‐salt” electrolytes. Recently the use of smaller and lighter [LiCl_2_]^−^ ions are reported by Kim et al.^[138]^ By the addition of choline chloride ((2‐hydroxyethyl)trimethylammonium chloride; ChCl), a large amount of LiCl (20 mol kg^−1^) was dissolved in water and [LiCl_2_]^−^ was the major anionic species. The low contents of free water molecules widen the electrochemical window of this solution and stage‐1 GIC of [LiCl_2_]^−^ was formed by electrochemical oxidation of graphite. They tested the electrochemical performance of graphite in various concentrations of electrolyte solutions and found that the capacity reached 110 mAh g^−1^, C_20_^+^LiCl_2_^−^ in 20 mol kg^−1^ LiCl +20 mol kg^−1^ ChCl (20L20C), as shown in Figure 17. They also achieved the intercalation of MgCl_3_^−^ into graphite.^[139]^

**Figure 18:**
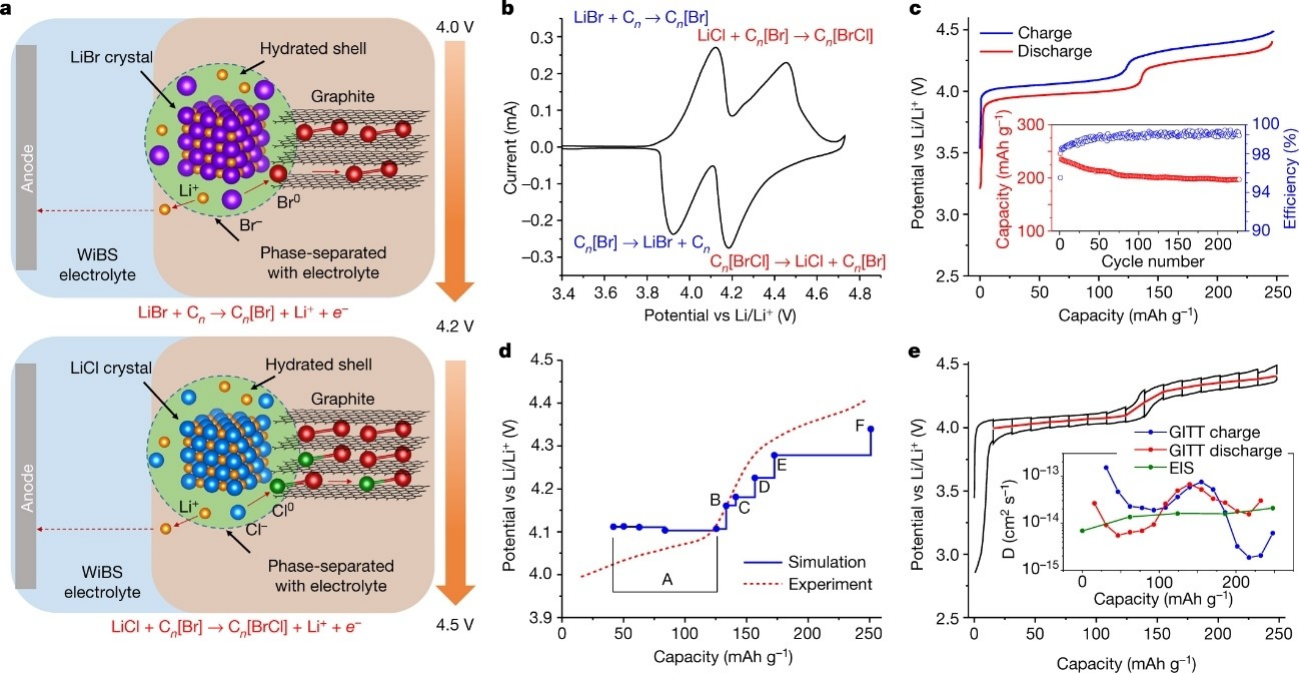
(a) Schematic of the conversion‐intercalation mechanism occurring in the (LiBr)_0.5_(LiCl)_0.5_‐graphite (LBC‐G) composite during its oxidation. (b) Cyclic voltammogram of the LBC‐G cathode. (c) Galvanostatic charge‐discharge profiles of the LBC‐G cathode, together with discharge capacity retention and Coulombic efficiency. (d) Comparison between the intercalation voltage predicted by density functional theory (DFT) simulations and the quasi‐equilibrium voltage curves obtained from GITT measurements. Reproduced from Ref. [140]. Copyright 2019, The Author(s).

In case of halogens such as chlorine and bromine, it has been difficult to intercalate them into graphite electrochemically probably because oxidation of chloride or bromide ions occur prior to the oxidation of graphite. However, Yan et al. have succeeded the reversible intercalation and de‐intercalation of bromine and bromine chloride by coating the surface of graphite with hydrated LiBr and LiCl.^[140]^ The charge‐discharge curve of coated graphite in LiTFSA‐LiCF_3_SO_3_ (LiOTf) containing gel type electrolyte is shown in Figure 18. During charging at 4.0–4.2 V vs. Li^+^/Li, the oxidation of Br^−^ to Br^0^ (near‐zero state) occurred and it was intercalated into graphite. Then, at 4.2–4.5 V vs. Li^+^/Li, similarly formed Cl^0^ is further inserted, resulting in the formation of stage‐1 GIC with a composition of C_3.5_[Br_0.5_Cl_0.5_]. The capacity reached a large value of 250 mAh g^−1^ based on the weight of composite electrode, (LiBr)_0.5_(LiCl)_0.5_C_3.7_. Liu et al. realized similar chemistry in 27.8 m ZnCl_2_ +0.8 m KBr and almost the same capacity was obtained.^[141]^ The storage of ICl in graphite is also possible in 120 m ChCl +30 m ZnCl_2_ +5 m KI solution and higher capacity of 291 mAh g^−1^, C_7.7_^+^ was delivered.^[142]^

Cai et al. investigated the charge‐discharge behavior of graphite in the aqueous solution of ZnCl_2_ and ZnBr_2_ (3 : 1 of molar ratio) mixture containing a small amount of Zn(CH_3_COO)_2_. Stage‐1 Br‐GIC was formed and a high capacity of 605.7 mAh g^−1^ (C_3.7_Br) was obtained.^[143]^ The capacity increased with the increase in the concentration of dissolved Zn salt and Coulombic efficiency was greatly improved at the same time.

### 4.2 Oxidation potential of graphite

**Figure 19:**
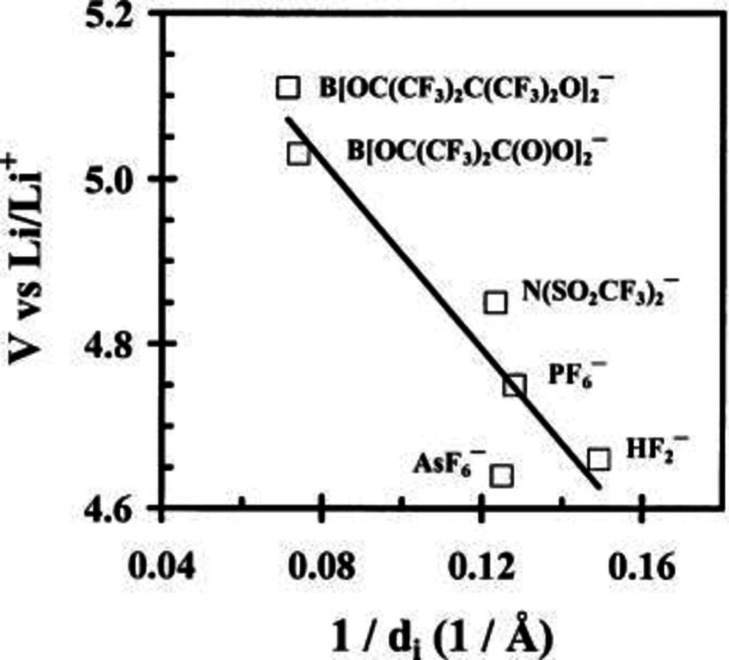
Relationship between oxidation potentials for stage 2 GIC and 1/di. Reproduced with permission from Ref. [144]. Copyright 2010 Elsevier.

As already mentioned, various anions are used in DIBs. In order to avoid the decomposition of electrolyte, that with a large electrochemical window should be used or it is necessary to operate with the low upper potential limits in order to improve Coulombic efficiency. Therefore, it is preferable if the intercalation potential of anions into graphite is lower. It has been reported that the oxidation potentials required to form a GIC vary for different intercalate anions. Özmen‐Monkul, and Lerner reported that the oxidation potentials to form electrochemically‐prepared stage‐2 GIC show approximately linear relation with the inverse of the gallery heights, 1/*d*i as shown in Figure 19.^[144]^ This is explained based on the simple thermodynamic model, where lattice enthalpies for GICs with similar composition are inversely proportional to the separation of ionic charges. This indicates that higher potential is needed to intercalate larger anions.

On the other hand, Beltrop et al. reported that the onset potential of the anion intercalation was in the order of BETA>FSA>FTSA>TFSA, which is not in the order of anion size (BETA>TFSA>FTSA>FSA).^[145]^ They suggest that ion pair formation and self‐aggregation in the electrolyte overrule the influence of the anion size. Haneke et al. reported the ratio of PF_6_^−^ and TFSA^−^ ions intercalated into graphite in 1.8 mol dm^−3^ LiPF_6_ +1.8 mol dm^−3^ LiTFSA/dimethyl carbonate based on the *ex situ* ^19^F NMR measurement.[^32^, ^145^] The ratio of intercalated TFSA^−^ increased as the increase in the cut‐off cell voltage, meaning that PF_6_^−^ ions are more easily intercalated into graphite than TFSA^−^.

Solvent molecules also play an important role to determine the intercalation potential. Zhu et al. reported the effect of the solvent on the electrochemical behavior of graphite in 1 mol dm^−3^ LiBF_4_/sulfolane (SL)+EMC solution. In the electrolyte solution without SL, no intercalation of BF_4_^−^ ions occurred while in those containing SL, BF_4_^−^ ions were successfully intercalated into graphite. The interlayer spacing of stage‐2 GIC of BF_4_^−^ anions was 0.74 nm when graphite was charged in 1 M LiBF_4_/SL, while it was a slightly larger value of 0.77 nm in 1 mol dm^−3^ LiBF_4_/SL containing EMC. They concluded that EMC molecules were co‐intercalated with BF_4_^−^ ions. They also suggested that solvent molecules with lower dielectric constants tend to solvate anions rather than lithium ions. They suggest two effects for the suppression of BF_4_^−^ intercalation into graphite, ion‐pairing with Li^+^ and solvation by EMC.^[146]^ More recently, Guan et al. revealed that the onset potential of intercalation of PF_6_^−^ into graphite is closely related to the solution structure of the electrolyte. They changed the proportion of free EMC and PF_6_^−^, and solvation number by adding 1,1,2,2‐tetrafluoroethyl‐2,2,3,3‐tetrafluoroprpylether to LiPF_6_/EMC electrolyte.^[147]^ The onset potential of intercalation became lower in the electrolyte containing lower proportion of free EMC and PF_6_^−^, and lower solvation number.

In the aqueous electrolyte, the difference of the intercalation potential greatly differs depending on the anions.^[148]^ The theoretical calculation reveled that the intercalation energies to form stage‐2 GICs for CF_3_SO_3_^−^ (OtF^−^), TFSA^−^ and BF_4_^−^ were −0.797, −1.147 and −2.445 eV, respectively. These values well explained the experimentally obtained charge‐discharge profiles except for that in the electrolyte containing OtF^−^. The authors explained this deviation is ascribed to the asymmetric shape of OtF^−^ which causes the high diffusion energy barrier in graphite. The large difference in the oxidation potential is ascribed to that in the hydration energy of anions. The hydration energy of BF_4_^−^ ions is much smaller than those of the others and therefore, the intercalation potential became lower.

### 4.3 Solvent/cation co‐intercalation

**Figure 20:**
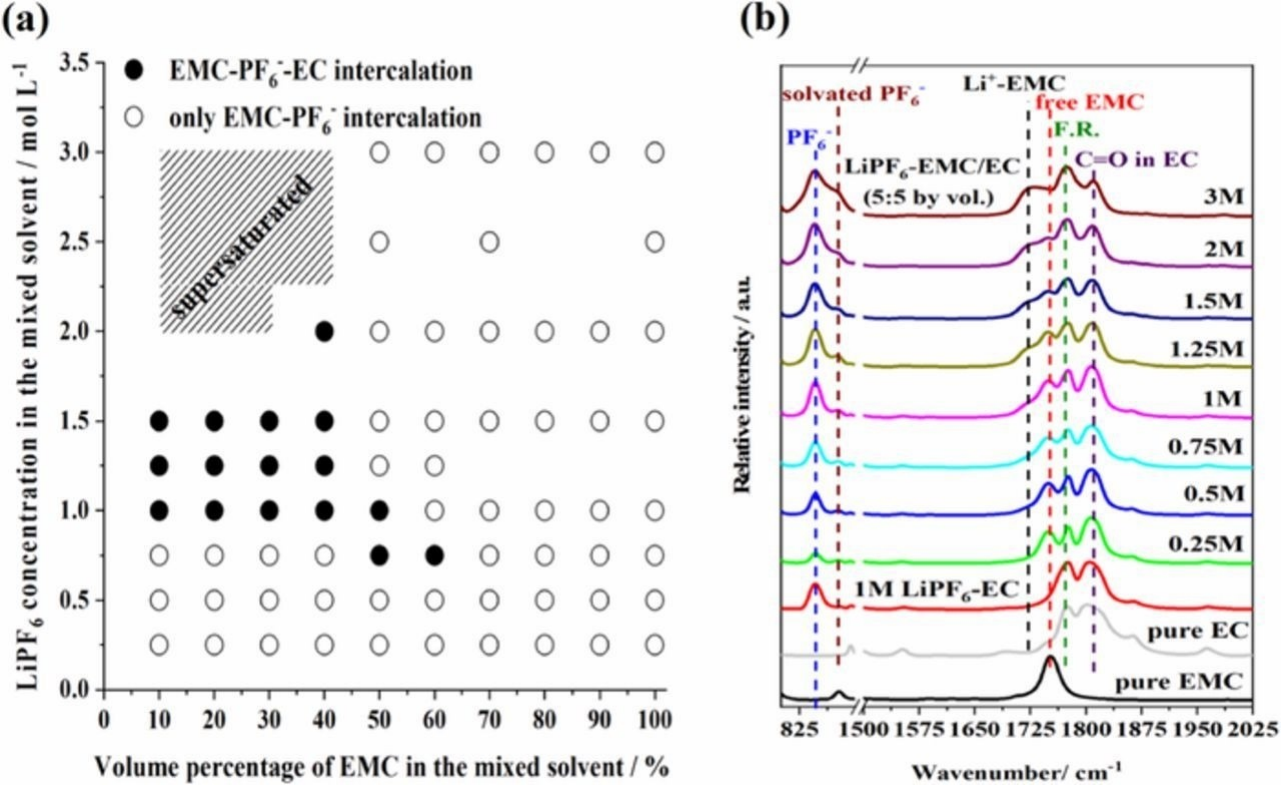
(a) Composition map of LiPF_6_‐EMC/EC solutions for intercalating species into graphite electrodes charged to 5.2 V vs Li/Li^+^, (b) FTIR spectra of LiPF_6_‐EMC/EC (5 : 5 by vol.) and the related solutions. Reproduced with permission from Ref. [162].Copyright 2019 IOP Publishing.

**Figure 21:**
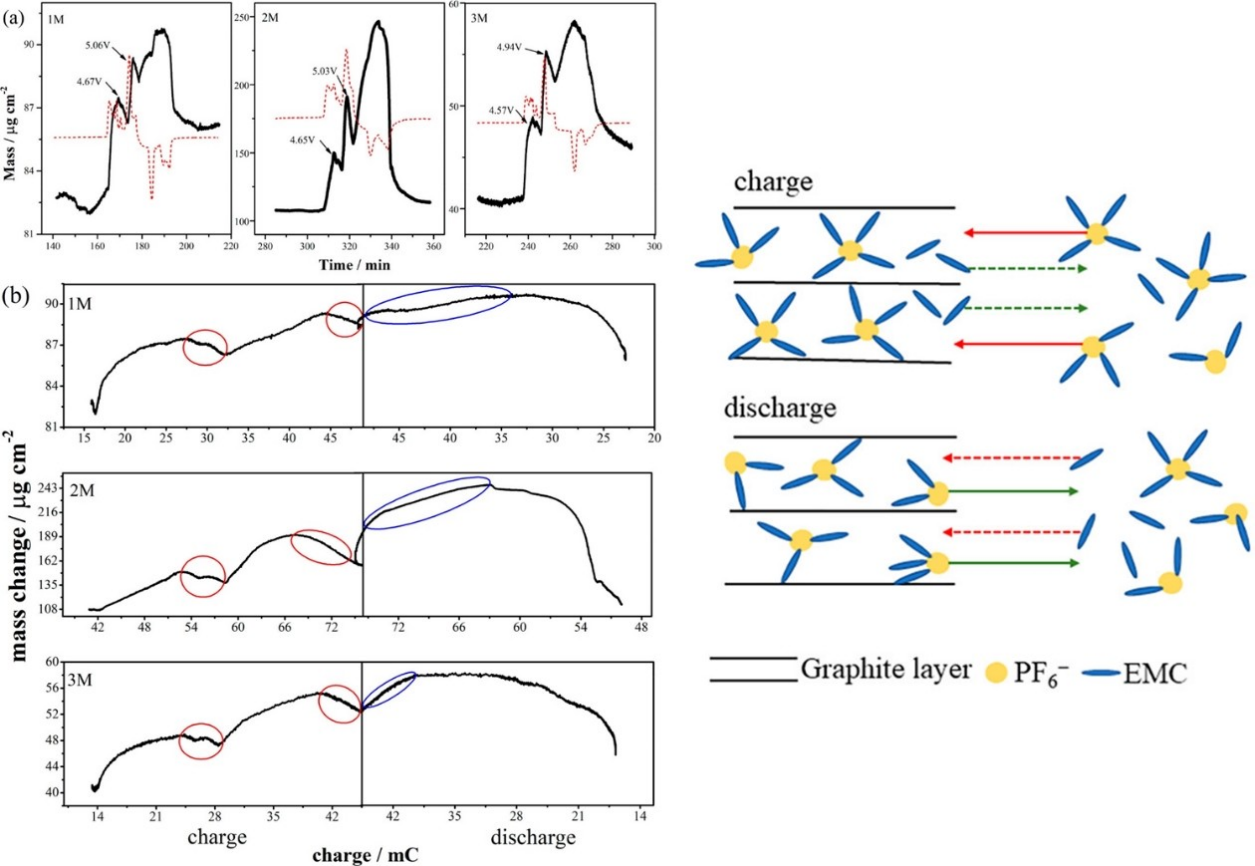
(a) Cyclic voltammograms and corresponding EQCM responses of a graphite electrode in LiPF_6_‐EMC solutions and (b) the relationship between the mass changes of graphite electrode and integrated charge of the CVs. Schematic illustration of counter flow of EMC molecules. Reproduced with permission from Ref. [146]. Copyright 2019 American Chemical Society.

Solvent co‐intercalation with cations, especially lithium ions into graphite is well known phenomenon. It occurs around 1 V vs Li^+^/Li and in ethylene carbonate (EC)‐based electrolyte solutions, following decomposition of the electrolyte results in the formation of surface film, so called solid electrolyte interphase (SEI). This avoids not only further decomposition of electrolyte but also co‐intercalation of solvent molecules. Therefore, intercalation of Li^+^ ions into graphite proceeds. On the other hand, co‐intercalation of propylene carbonate molecules results in the exfoliation of graphite, and they are continuously reduced, preventing the intercalation of Li^+^ ions. On the other hand, solvent co‐intercalation with anions have not been satisfactorily investigated and the effect of it on the electrochemical performance on graphite cathode is not so well clarified. In the electrolyte solutions, anions are also solvated even by the solvent with low dielectric constant such as EMC, which has been confirmed by Raman spectroscopy. Read has reported the co‐intercalation of EMC together with PF_6_^−^ ions based on the gravimetric and X‐ray diffraction data as mentioned above. The content of solvent was 0.6–1 molecule per one anion for fully charged graphite (C_20_PF_6_).^[27]^ Wang's group systematically investigated the co‐intercalation of solvents using various mixed solvents.[^149^, ^150^, ^151^, ^152^, ^153^, ^154^, ^155^, ^156^, ^157^, ^158^, ^159^, ^160^, ^161^, ^162^, ^163^, ^164^] For example, they investigated the co‐intercalation behavior of PF_6_^−^ in a mixed solvent of EC and EMC containing LiPF_6_.^[162]^ As shown in Figure 20, They found that EC, which has a larger donor number, preferentially and strongly solvates Li^+^, while both weakly solvate PF_6_^−^, and therefore EMC preferentially co‐intercalates at the high concentration region, where less EC molecules solvates PF_6_^−^. They also observed the decrease and increase in the weight during charge (intercalation) and discharge (de‐intercalation) by electrochemical quartz crystal microbalance (QCM), respectively, which indicates the counter flow of EMC molecules (Figure 21).

Co‐intercalation of cations together with anions into graphite cathode has been recently reported in 0.5 and 2.0 mol dm^−3^ Mg(TFSA)_2_/EMC electrolytes.^[165]^ As the increase in the concentration of Mg^2+^ ions in the electrolyte, the amount of co‐intercalated ones in the form of large‐sized anionic complexes ([Mg(TFSA)_3_]^−^) increased. This lead significant decrease in the sites viable for capacity contribution inside graphite galleries. The authors also found that the addition of adiponitrile as a co‐solvent lead to less Mg^2+^ co‐intercalation and increase in delivered capacity. They suggest that the low capacity observed for graphite cathode in electrolytes containing other multivalent metal cations (Ca^2+^, Zn^2+^) was also due to this co‐intercalation phenomenon.

### 4.4 Interfacial phenomena of graphite cathode

**Figure 22:**
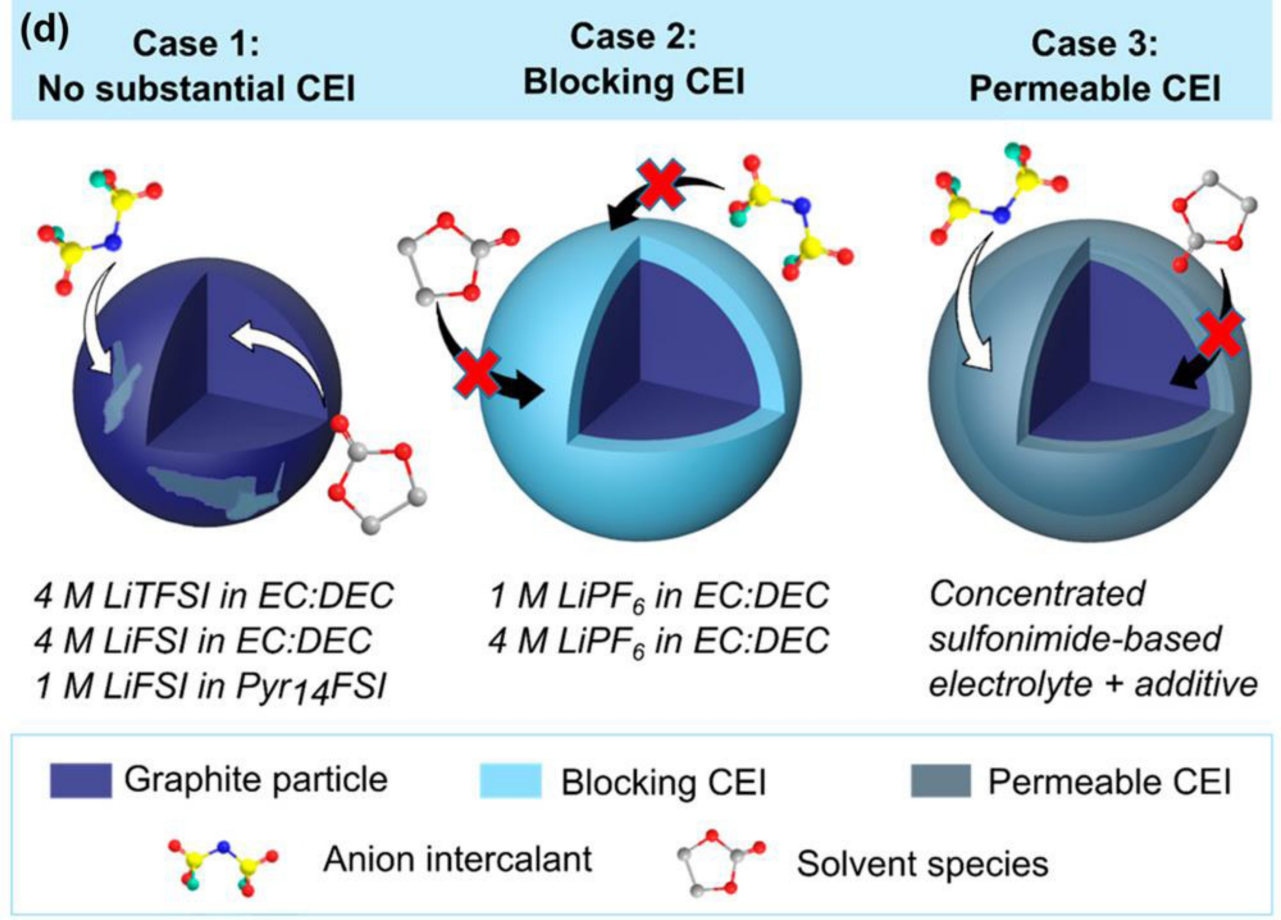
Schematic illustration of a graphite particle without CEI (1), with a blocking CEI (2), and with a CEI permeable to the anion intercalant (3). The third case is the most desirable, as this functional CEI allows for the insertion/deinsertion of the anion, while it blocks the solvent molecules from entering the graphite, causing side reactions and aggravated exfoliation. Reproduced with permission from Ref. [166]. Copyright 2021 American Chemical Society.

The other factors to determine the oxidation potential of graphite is kinetics of the reaction. The role of electrolyte solution has been also investigated in terms of cathode electrolyte interphase (CEI). The surface film formed as the result of the reductive decomposition of electrolyte solution at the surface of graphite anode is well known as solid electrolyte interphase, SEI. It has been indicated that similar surface film is formed at the surface of graphite cathode, and it greatly affect the electrochemical performance of DIBs. This is because the high working voltage of around 5 V vs. Li/Li^+^ leads to continuous side reactions, which results in the low Coulombic efficiency and poor cycling properties. Kotronia et al. have investigated the surface of graphite cycled in various electrolyte solutions.^[166]^ They concluded that in LiPF_6_/EC+DEC, a thick blocking CEI consisting of polyether, polycarbonate and decomposed salt (Li_x_PF_y_/PO_x_F_y_/Li_x_PO_y_F_z_) was formed, while in concentrated sulfonimide‐based electrolytes a thin and permeable one formed (Figure 22). Addition of fluoroethylene carbonate (FEC), tris(hexafluoro‐isopropyl)phosphate lithium difluorooxalateborate (LiDFOB) provide permeable CEI and, accordingly improve the Coulombic efficiency and cycle life.[^167^, ^168^, ^169^] It has been reported that the use of all‐fluorinated solvent is effective to form superior CEI.^[170]^ The resulting CEI was stable upto 5.2 V in graphite/Li cell and high Coulombic efficiency and cycle performance were achieved. Zhao et al. proposed the use of 3 mol dm^−3^ LiFSA FEC/methyl 2,2,2‐trifluoroethyl carbonate (FEMC) to form thin and permeable CEI and they also achieved high Coulombic efficiency, rate and cycle performances.^[171]^

An “anion‐permselective” polymer electrolyte with abundant cationic quaternary ammonium motif is developed by Jiang et al.^[172]^ It weakens the PF_6_^−^‐solvent interaction and thus facilitates PF_6_^−^ desolvation, preventing solvent co‐intercalation. The oxidation resistance of electrolyte was enhanced, and the Coulombic efficiency was improved. The electrochemical implantation of diethylenetriaminepenta(methylene‐phosphonic acid) on the surface of natural graphite particles generate an interphase that not only improves the antioxidative stability of electrolytes but also benefits desolvation of PF_6_^−^ anions.^[173]^ This also leads to fast‐charge and ultralong cycling performance of graphite. The deposition of inorganic substances such as Al_2_O_3_ and Li_4_Ti_5_O_12_ is also effective to improve the charge‐discharge properties.^[174]^

However, compared to the long‐established study of SEI on graphite anodes of lithium‐on batteries, CEI on the cathodes of DIBs has not yet been fully understood. Further elucidation of the function and models of CEI is expected in the future.

### 4.5 Kinetics of intercalation of anions

Fukutsuka et al.^[29]^ and Sagane,^[175]^ have reported the kinetics of anion (TFSA^−^, FSA^−^, CF_3_SO_3_^−^, ClO_4_^−^ and BF_4_^−^) intercalation into graphite using an electrochemical impedance technique. They concluded that the activation energy of interfacial anion transfer is about 12–28 kJ mol^−1^, which is much lower than that of Li^+^‐ion (50–60 kJ mol^−1^) and it is not influenced by solvation. In case of the intercalation of lithium, the surface of graphite is covered by SEI and desolvation of lithium ions should occur before the intercalation of lithium ions and this desolvation process determines reaction rate. On the other hand, anions are not strongly solvated, therefore, this process is not the rate‐determining one for electrochemical anion intercalation. Miyazaki et al. also investigated the kinetics of anion intercalation using electrochemical impedance technique in various concentrations of LiTFSA containing DEME‐TFSA ionic liquid.^[176]^ They found that the activation energy of interfacial anion transfer resistance was 39 kJ mol^−1^ independent of the concentration of LiTFSA and the pre‐exponential factor is correlated with the viscosity of the electrolyte. Sagane et al. also investigated the effect of the salt concentration of the electrolyte solution on the activation energy of interfacial anion transfer.^[177]^ The results showed that the activation energy increased significantly as the molar ratio of salt to solvent approached equimolar. It was found that this was due to the formation of contact ion pairs at high concentrations, which requires anion to break the electrostatic interaction with lithium ions for intercalation.

Another rate determining step is the diffusion of anions in the interlayer space of graphite. Ishihara et al. determined the diffusion coefficient of anions using galvanostatic intermittent titration technique (GITT) and electrochemical impedance spectroscopy (EIS) and obtained the value of around 10^−12^ cm^2^ s^−1^ for PF_6_^−^.^[178]^ Heckmann et al. also reported the diffusion coefficient value of TFSA^−^ to be 10^−15^.^[179]^ These values were comparable to that observed for Li^+^ diffusion in the cathode of lithium‐ion battery such as LiCoO_2_ and LiFePO_4_, 10^−10^–10^−14^ cm^2^ s^−1^. Ishihara et al. calculated the activation energy for PF_6_^−^ diffusion with nudged elastic band method of density functional theory calculation and found that it is slightly lower along the $\left\langle 100\right\rangle$ direction than along the $\left\langle 110\right\rangle$ direction.^[178]^

### 4.6 Other carbon cathodes

As shown above, the maximum capacity of graphite cathode seems to be around 111 mAh g^−1^ (C_20_A) except for the case where unusually high potential was applied or smaller halogens are utilized. Therefore, in order to increase the capacity of cathode, various carbon materials are explored. In case of the modification of graphite, a large capacity of 147 mAh g^−1^ ((PF_6_)C_15_) was reported when nanopores are introduced in it.^[180]^ The capacity of 132 mAh g^−1^ ((AlCl_4_)C_16.9_) for mechanically processed graphite flakes were reported and it reached 150 mAh g^−1^ ((AlCl_4_)C_14.9_), when it was charged by the constant current‐constant voltage charging protocol.^[135]^

#### 4.6.1 Soft carbons with different graphitization degree

One of the most frequently used parameters to describe the structure of carbons is graphitization degree. The structure of carbons changes depending on the heat‐treatment temperature. They transform to graphite when heated up to 3000 °C. The degree of graphitization is expressed by the parameters, *d*_002_ value, *L*_a_ and *L*_c_, etc. The *d*_002_ value is the distance between two adjacent carbon layers and, *L*_a_ and *L*_c_ are the crystalline size along a and c axis, respectively. In case of the anode of lithium‐ion battery, the effect of graphitization degree on the capacity has been already studied in detail.^[181]^ The effect of it on the amounts of stored anions have been also studied. Ishihara et al. investigated the electrochemical performance of various carbons with different interlayer spacings.^[182]^ The PF_6_^−^ de‐intercalation capacity increased with the decrease in it, which means that carbons with higher graphitization degree deliver higher capacity. Heckmann et al. also proposed that an increased area of “non‐basal plane” can increase the capacity in addition to the high graphitization degree.^[183]^ In case of AlCl_4_^−^ storage, similar results that carbons with higher graphitization degree deliver larger capacity are obtained.^[184]^ These results coincide with the previously reported intercalation behavior of non‐graphitized carbons. Recently, Shen et al. reported that petroleum coke‐based soft carbon delivers 96 mAh g^−1^ at a rate of 2 C and 72 mAh g^−1^ at a rate of 50 C.^[185]^ Yang et al. considered that more anions could be intercalated in locally ordered graphitized carbon, where interlayer interactions are weaker than in graphite and anion repulsion in the interlayer can be suppressed. Although it is necessary to discharge to considerably lower potentials, they demonstrated that PF_6_^−^ anion intercalation capacity of 232 mAh g^−1^ was actually obtained with a ketjen black cathode as the model material of locally ordered graphitized carbon.^[180]^

#### 4.6.2 Graphene based materials.

Recently, a number of papers on the performance of graphene‐based carbons have been reported. However, the main mechanism of the charge storage on these materials is adsorption of anions rather than intercalation of them. For example, graphene nanoribbon shows high AlC_4_^−^ storage capacity of 148 mAh g^−1^.^[186]^ Ejigu et al. reported that electrochemically exfoliated graphene can store large amounts of AlCl_4_^−^ (120 mAh g^−1^) and PF_6_^−^ (150 mAh g^−1^) ions. Addition of Co^2+^ ions during the electrochemical exfoliation of graphite in 0.2 mol dm^−3^ Na_2_SO_4_ aqueous solution provide graphene with less defects, which resulted in the large capacity.^[187]^

Reduced graphene oxide, rGO is also used as the cathode of DIBs.[^188^, ^189^, ^190^, ^191^] When GnO is reduced, most of the oxygen functionalities are removed, however, complete removal of them is difficult, and defects are also introduced. The degree of reduction, amount of defects, interlayer spacing, morphology, etc. of the resulting rGO greatly vary depending on the reduction conditions. It is also used as the electrode of electric double layer capacitor, EDLC in which anions are adsorbed at the surface of cathode for the storage of charge. No potential plateau observed during both charging and discharging of graphite appear for these materials.

**Figure 23:**
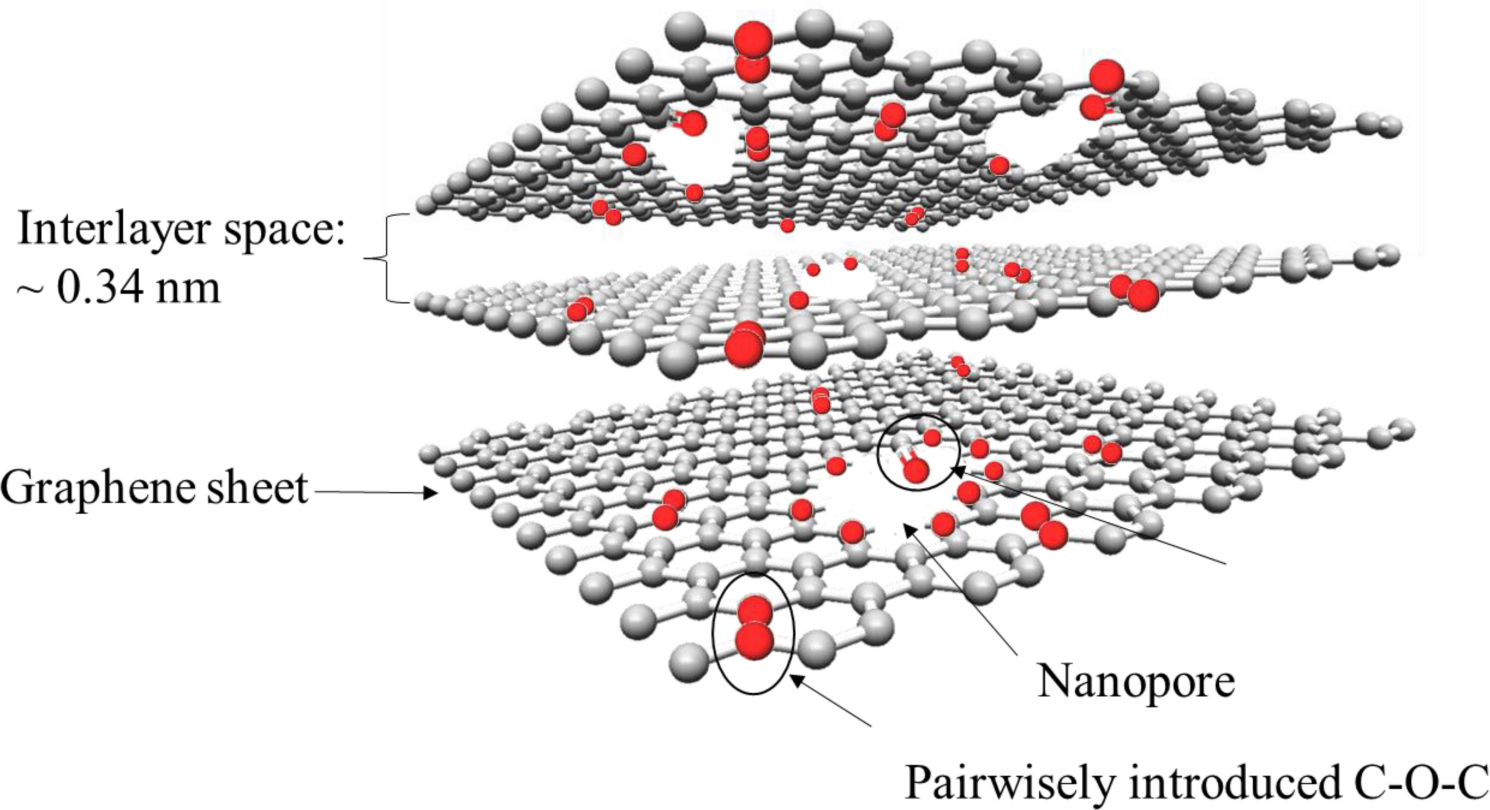
Structure model of graphene‐like graphite. Reproduced from Ref. [193]. Copyright The author(s).

**Figure 24:**
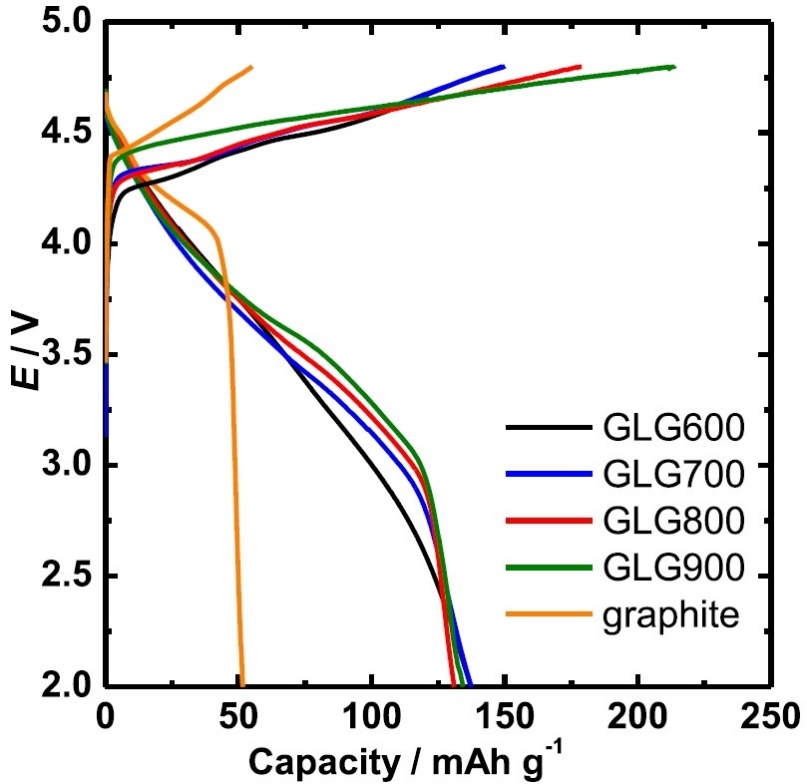
Charge‐discharge curves of graphene‐like graphite prepared at various temperatures in 3 M LiPF_6_‐EMC, together with that of graphite. Reproduced from Ref. [192]. Copyright The author(s).

On the other hand, by using GtO as a precursor and reducing it by heat treatment in an appropriate procedure, a carbon material with similar crystal structure and electrochemical ion storage behavior to graphite can be obtained. In this regard, Inamoto et al. have introduced graphene‐like graphite (GLG) as a novel carbon material for the cathode of DIBs.^[192]^ This material is synthesized by the thermal treatment of GtO.^[193]^ Unlike common rGO, where the stacking regularity of the carbon layers is almost entirely lost and the surface area is large, they are well preserved in GLG and the surface area is only slightly larger than that of raw graphite. Oxygen atoms are introduced within the graphene layers in the form of C−O−C as shown in Figure 23.^[193]^ This material was first developed for the anode of lithium‐ion battery and, shows high capacity and superior rate performance.^[194]^ It is basically similar material to partially reduced GtO[^195^, ^196^] or pillared graphite^[197]^ developed for the electrode of EDLC. Figure 24 shows the charge‐discharge curves of GLG prepared at various temperatures in 3 M LiPF_6_/EMC, together with that of graphite. During charging a plateau was observed at lower potentials than that of graphite. While graphite shows only 50 mAh g^−1^ with a relatively low cutoff potential of 4.8 V vs Li^+^/Li, stage‐1 GIC was formed and the capacity reached 137 mAh g^−1^.^[198]^ The capacity further increased to 149 mAh g^−1^, when FSA^−^ was used as anions. The density functional theory calculation indicated that the introduction of oxygen‐containing functional groups into graphite emerges new bands just at or slightly lower than the Fermi level.^[199]^ Since electron are withdrawn during anion intercalation, the bands enable anion intercalation at lower potential compared to graphite. As a result, when charged to the same potential, the anions can be intercalated into GLG up to lower stages than graphite, leading to a higher capacity. Recently, it was demonstrated that dual GLG battery, which employs GLG as anode and cathode and 3 M LiFSA/EMC as electrolyte solution, well works with even low cut‐off voltage of 4.6 V.^[200]^ They also reported the intercalation/de‐intercalation of fluorine into/from GLG when it is used as the cathode of all solid‐state fluoride shuttle batteries.^[201]^ Thus, recent progress in carbon‐based cathode material largely improved the capacity of cathode, which is one of the drawbacks of DIBs with graphite cathode. This has made it possible to achieve performance comparable to conventional LIB cathodes in terms of capacity per weight. Further research is expected to lead to the practical realization of DIBs in the future.

## 5 Summary and Outlook

In this review, fundamental aspects of the electrochemical intercalation of anions into graphite have been first summarized, and then described the electrochemical preparation of covalent GICs and application of graphite as the cathode of dual‐ion battery.

Various anions can be intercalated into graphite, and the content of them and the structure of the resulting GICs greatly vary depending on the properties of them. Electrochemical overoxidation of anion GICs provides graphite oxide and covalent fluorine GICs. In case of the preparation of graphite oxide, it is usually obtained in concentrated oxoacid aqueous solutions such as sulfuric acid, perchloric acid, etc. The introduced oxygen functionalities into graphite seem to be derived of water molecules existing in the electrolyte solutions, therefore, electrolyte solutions other than oxoacids, such as aqueous HBF_4_ one can be also used. The concentration of electrolyte solution is not necessarily high, if high voltage was applied to graphite. A large amount of graphite oxide has been prepared at high yields in appropriate electrolyte solutions by applying sufficient voltage or current to graphite. The reaction mechanism of overoxidation has not been fully understood yet, therefore, in order to produce graphite oxide at industrial levels, further investigation of it would be needed, together with the design of the improved electrochemical cells. When fluorine‐GIC was overoxidized in an aqueous hydrofluoric acid solution, carbon‐fluorine covalent bonds are first formed, providing covalent fluorine GIC, C_x_F. Further oxidation of it resulted in the oxidized C_x_F in which covalently bonded fluorine and oxygen atoms are uniformly distributed on graphene layer.

Concerning the application of graphite for the cathode of dual‐ion battery, it stably delivers about 110 mAh g^−1^ (C_20_^+^) of reversible capacity in usual organic electrolyte solutions, containing LiPF_6_, LiTFSA, etc. The combination of anion and solvent as well as the concentration of the anions in the electrolyte solutions greatly affect the electrochemical performance of graphite cathode such as oxidation potential, rate capability, cycling properties, etc. Since there still enough space to accommodate more anions in the interlayer space of graphite, optimization of electrolyte solutions seems to increase the capacity of graphite cathode. The interfacial phenomenon is also important, and fundamental studies of charge transfer resistance, anion diffusion coefficient, and surface film formation behavior have also been summarized. The use of smaller anions, such as AlCl_4_^−^, Br^−^ can increase the capacity of graphite cathode in appropriate electrolyte solutions where the decomposition of them is effectively avoided. Several efforts on the structural modification of graphite and development of electrolyte solutions in which graphite cathode delivers higher capacity were also described. Apart from these efforts, the other important issues such as swelling of the cathode originating from the larger expansion of interlayer spacing than that of the anode, cross‐talk reactions, etc. should be also addressed for the practical application of DIB. The development of binders[^202^, ^203^, ^204^] and current collectors,^[205]^ precycle of anode,^[206]^ etc. have been conducted for these purposes, which will be summarized elsewhere.

It would be very nice if this review can give the readers the insight to further improvement of the preparation procedure of graphite oxide and the performance of graphite cathode in dual‐ion batteries.

## Conflict of interests

The authors declare no conflict of interest.

6

## Biographical Information

*Prof. Yoshiaki Matsuo is a professor in the Department of Applied Chemistry at University of Hyogo (Japan) since 2017. He received the Ph.D. in Engineering from Kyoto University in 1995 for his work on carbon‐fluorine materials. His current research interests are chemical modification of layered materials, especially graphite oxide and application of the resulting materials for various energy storage devices such as dual‐ion, sodium ion, all‐solid‐state lithium ion and fluoride shittle batteries*.

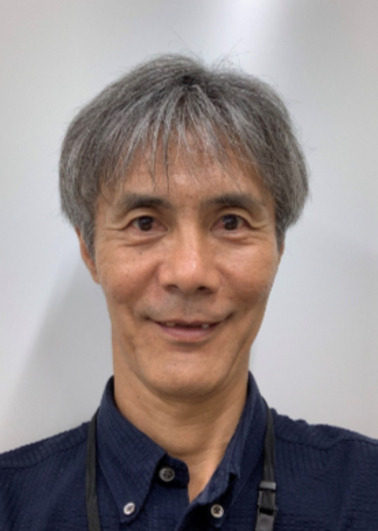

## Biographical Information

*Dr. Junichi Inamoto is an assistant professor at the Department of Applied Chemistry, University of Hyogo since 2017. He obtained his Ph.D. in Engineering from Kyoto University in 2017 for his work on surface analysis of cathode materials of LIBs using thin‐film electrodes. His research interests include fundamental analysis of various rechargeable battery materials. He uses model electrodes in combination with various electrochemical methods, spectroscopic analysis, and theoretical calculations to elucidate the phenomena occurring at the interface and inside the active materials*.

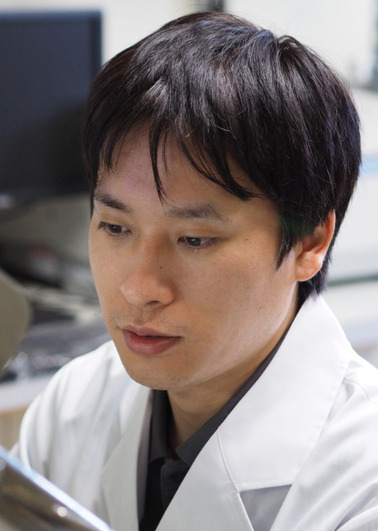

## Biographical Information

*Dr. Akane Inoo is a specially appointed assistant professor at the Department of Applied Chemistry, University of Hyogo since 2020. She received a Ph.D. in Engineering from Kyoto University in 2020. She was engaged in research on magnesium deposition/dissolution reactions for magnesium secondary batteries and the interfacial lithium ion transfer through surface films on graphite electrodes at Kyoto University. From 2020, she works on the development of carbon‐based electrodes for next‐generation batteries such as fluoride‐ion batteries and aqueous zinc‐metal batteries*.

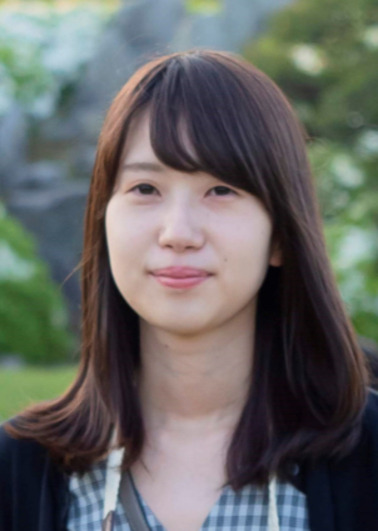

## Data Availability

Data sharing is not applicable to this article as no new data were created or analyzed in this study.
